# Status of health care waste management plans and practices in public health care facilities in Gauteng Province, South Africa

**DOI:** 10.1186/s12889-023-15133-9

**Published:** 2023-02-06

**Authors:** Tumisang Ramodipa, Koos Engelbrecht, Ingrid Mokgobu, Daniel Mmereki

**Affiliations:** 1 Department of Environmental Health, Tshwane University of Technology, Staatsartillerie Rd, Pretoria, 0183 South Africa; 2 Faculty of Health Sciences, School of Public Health, University of Witwatersrand, Johannesburg, 2193 South Africa

**Keywords:** Gauteng Province, Health care waste, Environmental, Integrated approach, Health, Safety

## Abstract

**Background:**

Health care waste management is a challenge due to the composition of the waste generated within a health care facility, of which 85% is domestic waste, and at least 15% is hazardous waste or health care risk waste (has been in contact with blood, body fluids or tissues from humans and could cause disease). In this study, we evaluated the status quo of health care waste management plans (HCWMPs) and practices in public health care facilities in Gauteng Province, South Africa.

**Methods:**

A situational analysis was employed in health care facilities (HCFs) that generated more than 20 kg (*N* = 42) of health care risk waste (HCRW) per day. Data was collected from officials responsible for the management of health care waste using a self-administered questionnaire whilst Chief Executive Officers/ managers of the HFCs were interviewed.

**Results:**

The results showed that most (79.0%) of the health care waste officers (HCWOs) as well as management (84.6%) agreed to have HCWMPs in place. The majority (76.9%) of the HCFs have a dedicated person appointed to manage health care waste with the majority (67%) being environmental health practitioners. According to management, only 30.8% have formally appointed an integrated HCW committee. Only 11.7% of the HCWOs are guided by the Occupational Health and Safety Act to develop their HCWMPs with only 20.5% with health care waste minimisation strategies in place.

**Conclusion:**

The study concluded that there is limited integration of HCWMPs as inadequate health and safety aspects, environmental pollution as well as community participation was reported. The novelty of the study is to contribute to a body of knowledge, information on the establishment of an effective health care waste management system in public health care facilities and for decision-making purposes.

**Supplementary Information:**

The online version contains supplementary material available at 10.1186/s12889-023-15133-9.

## Introduction

Health care facilities (HCFs) sustain and improve the quality of life, which ultimately lead to increased quantities of health care waste (HCW) generated from these facilities [1]. Health care waste (HCW) has become an issue of increasing global concern due to increased urban populations, quantities, and composition of this waste. The health and environmental implications are mounting in urgency, and this is a daunting challenge in developing countries [2]. In developing countries such as South Africa, HCW has not received sufficient attention due to the competing needs with other sectors of the economy for the very limited resources available. Health care waste management is not being given the high priority it deserves [3].

The World Health Organisation describes HCW as all the waste generated within the HCFs, of which about 85% is domestic waste which comprises paper, plastic, packaging etc*.* At least 15% of HCW is hazardous waste or health care risk waste (HCRW), which is defined as waste that has been in contact with blood, body fluids or tissues from humans and can cause disease [4]. This waste stream should be managed within the HCFs from the point of generation to treatment/disposal. All employees managing and handling health care waste must be trained on correct identification or classification, segregation, containerisation, and storage of the waste to minimise environmental health risks [5]. Effective implementation of appropriate waste management practices depends on the national regulations, occupational health & safety legislations, internal policies, qualifications, and competence of all those managing and responsible for the waste. This should be accompanied by provision of sufficient training programs and protective measures by HCFs to all relevant personnel, and adequate financial support and effective administrative monitoring by the respective authorities [6].

Currently, the Gauteng Health Care Waste Management Regulation [7] is the statute used to manage HCW within HCFs in the province. This includes the guidance on the development of a health care waste management plan (HCWMP) if the HCF generates more than 20 kg of health care waste per a day [7]. In addition to that, the Occupational Health and Safety Act [8] and novel environmental legislation such as the National Waste Strategy [9] needs to be acknowledged within the HCWMPs to ensure legal compliance from point of HCRW generation to the final disposal site. On the same note, sustainable environmental practices, impact on the environment and exposure of workers (occupational health and safety) is not fully given highest priority in health care waste management plans. However, the White Paper on Integrated Pollution and Waste Management [10] promotes the shift from the uncoordinated pollution prevention and control of waste management to integrated pollution and waste management. This holistic and integrated strategy is aimed at pollution prevention and minimization of waste at the source, managing the impact of pollution and remediating damaged environments. This will give effect to the Constitutional rights of Section 24 (a) of the constitution of South Africa [11], which stipulates that all people have the right to a clean and healthy environment.

A study in the Eastern Cape found that there is no clear strategic document from any national authorities that guides the management of HCW. Poor waste management practices and illegal dumping in unapproved sites were reported. The researcher recommended that a policy should be developed to assist in the management of HCW [12]. To meet this disparity, the researcher evaluated the status of HCWMPs in public health care facilities, Gauteng Province. The novelty of the research is to add information to the existing literature and help decision-makers to formulate or develop an effective system. In view of this, the objectives of the study were to: (1) conduct a situational analysis of the status quo of HCWMP and practices applied in the HCFs, (2) provide recommendations to improve the current policies and (3) to evaluate the possibility of developing an integrated HCWMP for the major generators.

## Methods

### Study design and study settings

In this study, a situational analysis was employed to evaluate the integration of the HCWMPs in public health care facilities. A situation analysis is a useful process of critically evaluating the internal and external conditions that affect an organization. It was beneficial to this study as it gives a comprehensive view of the current situation and identifies the current strategies and activities in place to overcome the problem [13]. Although situation analysis has been applied in other fields such as economics, it has also been extensively explored in other fields such as waste management. For instance, Mohamed et al. [14] used situation analysis for biomedical waste management in primary health care units of Ismailia district in Egypt. Gabela [15]) employed situation analysis for health care waste management in public clinics in the iLembe District, KwaZulu Natal, South Africa. In this study, we employed situation analysis to study the integration of the HCWMPs in public health care facilities in Gauteng Province, South Africa.

The study was carried out in Gauteng Province which is one of the nine provinces in South Africa where about 25.3% of South Africa’s population resides, accounting for 14,278,700 (Gauteng Department of Health: Contract circular GT-GDoH 168. Internal document, unpublished) of the country’s population. Gauteng Province comprises five districts namely: City of Johannesburg; City of Tshwane; Ekurhuleni; Sedibeng and West Rand. The Gauteng Department of Health has a total of 39 hospitals, 400 clinics, 11 forensic pathology service departments, 36 emergency medical service stations, four laundries, six nursing colleges and various funded non-governmental organisations (Gauteng Department of Health: Contract circular GT-GDoH 168. Internal document, unpublished).

The study looked at major generators which are HCFs that generate more than 20 kg of HCRW per day [7]. Table 1 below is a list of major generators in the Gauteng Province [16].

**Table 1:** List of major generators in Gauteng Province [16]

District	Type of facility	Number
**Johannesburg** *N* = 16	Hospitals	7
	Community Health Centre	7
	Clinic	1
	Forensic and pathology services	1
**Tshwane** *N* = 12	Hospitals	10
	Dental Hospitals	2
**Ekurhuleni** *N* = 7	Hospitals	6
	Community Health Centre	1
**Sedibeng** *N* = 4	Hospitals	3
	Community Health Centre	1
**West Rand** *N* = 3	Hospitals	3

Total *N* = 42

### Study population and sampling

This study included HCFs that generate more than 20 kg (*N* = 42) of HCRW per day. The contract circular [16] comprises of all the HCFs in Gauteng Province with average monthly HCRW generation volumes from April 2013 to March 2014. For this study, the contract circular was used to determine the total population (N) of all the HCFs producing 20 kg or more of HCRW per month in the entire province. The total population is 42.

The questionnaire was administered to forty-two (42) HCW officers and 35 (83.3%) responded. One HCW officer responsible for the HCWP was selected per HCF to participate in the study. For this study, the total population was used. Utilising the whole population provides deep insights into the topic of interest by reaching a wider coverage of the population of interest. This reduces the risk of missing potential insights from members that are not included.

Concerning the CEOs or facility managers, a stratified random sampling technique was used to select 22 HCFs out of a total of 42 (50% of total facilities in Gauteng Province) for interviews. This type of sampling was employed as the total population can be partitioned into subpopulations as the volume of HCRW within the various HCFs varies in kg from very large to just over 20 kg per day [17]. To obtain a proportional representation by district, all sampled HCFs were selected based on generation of more than 20 kg a day of health care waste. The type of facility and the size of health care waste were also considered when doing the random selection. Thirteen out of the 22 responded to the interview schedule (response rate of 59.1%). Table 2 below illustrates the study population used for the CEOs or managers.

**Table 2:** The total facilities used for the structured interview schedule (*n* = 22) to interview the CEO or facility manager

Number	District	Total population (N)	n( 50%)	Final sample (*n* = 22)
1	Johannesburg	16	8	8
2	Tshwane	12	6	6
3	Ekurhuleni	7	3.5	4
4	Sedibeng	4	2	2
5	West Rand	3	1.5	2
	**Total**	**42**	**21**	**22**

### Data collection tools

A questionnaire survey was used by the researcher to collect data from the HCFs. The researcher achieved this by disseminating the questionnaires to the respondents at the HCFs and allowed ample time for completion. Individual holding the responsibility of executing the overall responsibility of health care waste management were requested to complete the questionnaire. A structured interview was used to test opinions of the various managers at different HCFs [18]. This provided an opportunity for managers to express their views on strategic issues related to HCW at their health care facilities [19]. The interview was mostly conducted with the CEOs or managers responsible for the overall management of the facility. A detailed interview guide was used during a structured interview. This enabled the researcher to have control over the topics and the format of the interview.

A semi-structured questionnaire for the health care waste officers was developed based on existing data (national and international) and information on applicable health & safety and environmental legislation relating to health care waste management plans. The questionnaire was successfully piloted on independent knowledgeable professionals who are in the field of HCW to assess clarity of the operational questions and ensure that they are structured accordingly with the time schedule to provide the correct data required for the study.

### Analysis of data

For this study, a quantitative method was followed for analysis of the numerical data. Statistical analysis was conducted using Statistical Package for the Social Sciences (SPPS) version 25 for all questionnaires and interviews. Descriptive analyses such as frequencies, percentages and means were used to summarize data. Data (graphs, pie charts, tables and cross tabulation) was populated, and determination of trends was undertaken. The statistician assisted with the interpretation of the results.

## Results

### Demographic profiles of the health care waste officers and management

The demographic profiles of the respondents that completed the questionnaires are illustrated in Table 3. The findings of the study indicated that more females (82.4%) than males (17.6%) participated. The majority (47.1%) had degrees and were practicing as Environmental Health Practitioners (EHPs) (55.9%). They were environmental health practitioners (55.9%) and Infection prevention and control nurses (23.5%). The majority of the HCFs were at the City of Johannesburg and the City of Tshwane, accounting for 29.4% each, followed by West Rand District (8.8%), having the least respondents. With regards to the type of HCF, district hospitals (35.3%) had the highest percentage of reporting followed by regional hospitals (29.4%).

**Table 3:** Demographic profiles of the health care waste officers

Demographics	Frequency (n)	Percentage (%)
**Gender**		
Male	6	17.6
Female	28	82.4
**Highest level of education completed**		
Matric	2	5.9
Post matric diploma	11	32.4
Post matric degree	16	47.1
Postgraduate	3	8.8
Certificate	2	5.9
**Job title of respondents**		
Environmental Health Practitioner	19	55.9
Infection Control Nurse	8	23.5
Health Care Waste Officer	3	8.8
Occupational Health and Safety Nurse	2	5.9
Nursing Manager	2	5.9
Other	2	5.9
Health care waste officer/ Infection control prevention and Occupational health nurse	1	2.9
Procurement	1	2.9
**Districts of the health care facilities**		
Ekurhuleni	7	20.6
City of Johannesburg	10	29.4
Sedibeng	4	11.8
City of Tshwane	10	29.4
West Rand	3	8.8
**Type of health care facilities**		
Tertiary Hospital	3	8.8
Regional Hospital	10	29.4
District Hospital	12	35.3
Community Health Centre	5	14.7
Maternity and obstetrics Unit	1	2.9
Academic Hospital	3	8.8

Table 4 depicts the educational background of the management interviewed. Most of the respondents (38.5%) had Health Science Degrees, followed by those with Health Sciences postgraduate Degrees (23.1%).

**Table 4:** Educational background of the management

Education	Frequency (n)	Percentage (%)
Medical Doctor	1	7.7
Health Sciences Post Degree	3	23.1
Health Sciences Degree	5	38.5
Other	4	30.8
Master in Public Health	1	7.6
Master in Public administration	1	7.7
B-tech Nursing	2	15.4

### Status of health care waste management plans

The majority of the HCWOs (97.1%) confirmed that they have received HCW training. All the HCWOs were familiar with the Gauteng Health Care Waste Regulations. More than half (53.8%) of management reported having HCW included in their job descriptions.

**Fig. 1:**
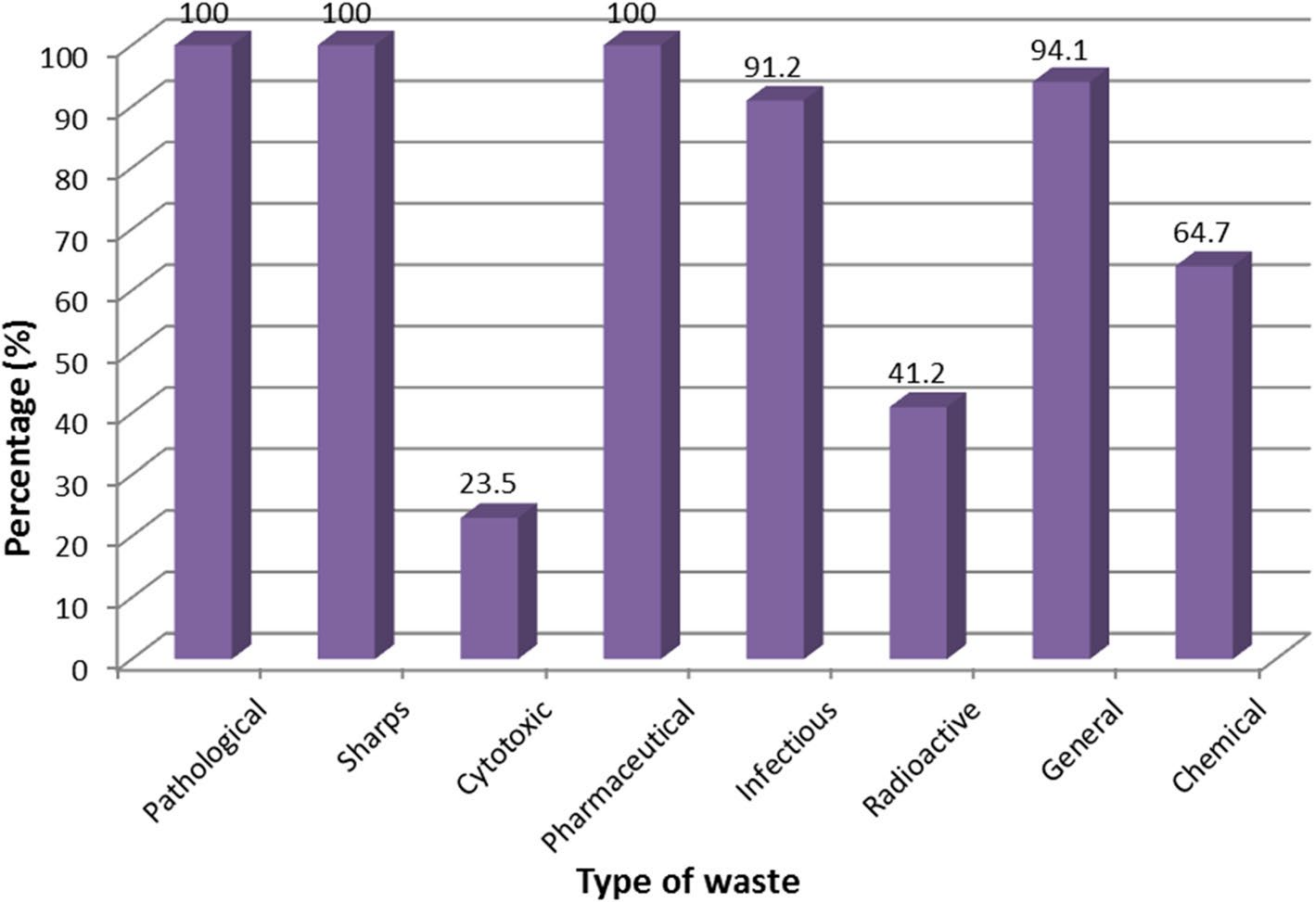
Types of HCW generated in the health care facilities

The question “what type of HCW does your HCF generate?” was asked to HCWOs and the following type of waste was listed as waste generated at all HCFs: pathological, sharps, cytotoxic, pharmaceutical, infectious, radioactive, general and chemical waste respectively as depicted in Fig. 1.

The majority of the HCWOs (76.5%) and management (76.9%) indicated that their facility generated more than 20 kg of HCW per day. The HCWOs (79%) reported that they have a HCWMP in place. Table 5 presents the roles of management in the implementation of the health care waste management plans (HCWMPs). Less than half of the management (38.4%) indicated that they ensure that the HCWMPs are executed, with only 15.3% reporting that they ensure that the HCWMPs are completed and approved.

**Table 5:** Role of management in the implementation of the health care waste management plans

Role	Frequency (n)	Percentage (%)
Ensure that it is executed	5	38.4
Developed the plan	3	23
Ensure that it is completed and submitted to the relevant authorities	3	23
Ensure that it is completed and approved	2	15.3

With reference to the operational and strategic decisions on HCWMPs by HCWO and management, only 17.6% of the HCWOs reported that they revert to the HCWMP when operational decisions are made. More than half (53.8%) of management uses the HCWMPs to make strategic decisions.

Health care waste officers agreed that the HCWMP is a necessary tool that promotes the management of HCW (70.6%) and all the management were in agreement that HCWMP is a necessity. Management confirmed that there are dedicated personnel to manage HCW at the HCFs (76.9%). Of the 23.1% who reported that they do not have a person that manages HCW in the HCFs, 8% indicated that it was due to budget constraints.

Table 6 illustrates the job title of the persons responsible for the management of HCW at the HCFs. The majority (67.6%) of the professionals that are managing HCW are environmental health practitioners and the least professionals that are involved in HCW management are occupational health coordinators, accounting for 8.8%.

**Table 6:** Individuals responsible for management of health care waste at the health care facilities

Job title	Frequency (n)	Percentage (%)
Environmental Health Practitioner	23	67.6
Infection Control Nurse	10	29.4
Health Care Waste Officer	15	44.1
Occupational Health and safety Coordinator	3	8.8
Nursing Manager	5	14.7

The following question: “is there a designated person/s responsible for collection and storage within the HCF”? was asked. The majority of the HCWOs agreed that there is a person (79.4%) who is responsible for HCW collection and storage within the health care facility. They also indicated that training is provided (79.4%). The designation of the person responsible for the waste collection were general assistants (40.7%) followed by cleaners (37%) and health care waste collectors were the least reported (11.1%).

### Integration of health care waste management plans into business operations

Table 7 below presents the various legislation and/or guiding documents used to develop the HCWMP as reported by the health care waste officers.

**Table 7:** Legislation/guiding documents used to develop health care waste management plans

Legislation/ guiding documents	Frequency (n)	Percentage (%)
Gauteng Health Care Waste Regulation	15	44.1
National Environmental Management: Waste Act	9	26.4
National Environmental Management Act	5	15.7
Occupational Health and Safety Act	4	11.7
National Health Act	2	5.8
Cradle to grave principles	1	2.9
National core standards	1	2.9
Infection Prevention and Control policy	1	2.9
World Health Organisation guidelines	1	2.9
SANS 10,248	1	2.9
Road traffic Act	1	2.9
National waste strategy	1	2.9
National Environmental Management: Air Quality Act	0	0
Regulations Regarding the General Control of Human Bodies, Tissue, Blood Products and Gametes: Amendment	0	0

The findings of this study indicate that the most (44.1%) used document was the Gauteng Health Care Waste Regulation of 2004, followed by the National Environmental Management: Waste Act (26.4%). The National Environmental Management accounted for 15.7% while the Occupational Health and Safety Act accounted for 11.7%. Surprisingly, none of the respondents reported to have used the National Environmental Management: Air Quality Act and the Regulations Regarding the General Control of Human Bodies, Tissue, Blood Products and Gametes: Amendment.

A question on the appointment of a HCW committee was asked. A lower percentage of HCWOs reported that an integrated HCW committee (24%) has been formally appointed with less than half (47.1%) of the HCFs having no HCW committee in place. With regards to the management, 30.8% reported that they have formally appointed an integrated HCW committee, with majority (61.5%) indicating that they have not appointed an integrated HCW committee at their health care facility. Respondents that reported on the existence of an integrated HCW committee (24%) were requested to list a maximum of eight professionals that make up the committee. From Table 8, infection prevention and control nurses (7) were the most recorded professionals, followed by environmental health practitioners and occupational health coordinators (6). The head of the pharmacy, HCW officer-professional nurse, professional nurses, environmental officer, head of radiology, skills and development, porters, caretakers, and facility manager are listed as the least presented.

**Table 8:** Details of professions of the integrated health care waste committee members

Profession	Member 1	Member 2	Member 3	Member 4	Member 5	Member 6	Member 7	Member 8	Total
Head of Pharmacy	1								1
Environmental Health Practitioner	1	1	1				1	2	6
HCW Officer-Professional Nurse	1								1
Health Care Waste Officer	1		1						2
Nursing manager	1	1					1		3
Infection Prevention and Control Nurse	3	1	1	1			1		7
Logistics		1			1				2
Occupational Health and Safety coordinators		3	1			1		1	6
Professional Nurse		1							1
Clinical Manager			1						1
Environmental officer			1						1
Quality Assurance Coordinator			2	1	1	1			5
Pharmacy Manager				2					2
Cleaner				2		1			3
Procurement				1			2		3
Finance					1	1			2
Nursing Operational Manager					1	1			2
Head of Radiology					2				2
Waste Handler					1				1
Skills and Development						2			2
Porter							1		1
Care Taker								1	1
Facility Manager								1	1

The following question: “Is there a system that ensures retrievability and accessibility of documents”? was asked. All the management reported that there is a system that ensures retrievability and accessibility of HCW documentation compared to 77.7% reported by the health care waste officers. Filing system was available (31%), and files were accessible through the reception (31%) and copies were made on every important document, which accounted for 31%. The following question: “Does the HCWMP make provision for emergency conditions”? was asked and 70.6% of the HCWOs reported that the HCWMP makes provision for emergency conditions related to health care waste, but 32% of the HCWOs and 31% of management reported that the motivations were submitted if the HCF needs budget when addressing shortfalls pertaining to health care waste.

### Integration of occupational health and safety into health care waste management plans

The majority of the HCWOs (79.4%) reported that the HCWMPs include health and safety hazards and risks. All the HCWO indicated that there is a procedure in place for reporting injuries related to health care waste. Moreover, HCWOs (70.3%) indicated that the health and safety measures are reviewed whilst 2.9% indicated that they are not being reviewed and 22.2% reported that they are uncertain if they get reported.

Table 9 provides information on the requirement in the Occupational Health and Safety Act [8] on reporting of incidents. A total of 12 (44.4%) respondents indicated reporting after every incident, followed by annually (11.1%).

**Table 9:** Revision frequency of the health and safety incidents

Revision frequency	Frequency (n)	Percentage (%)
After every incident	12	44.4
Annually (once a year)	3	11.1
Bi-annually (every 6 months)	2	7.4
Every 2 years	2	7.4

A question on whether health and safety aspects are complied with regarding HCRW was asked. The HCWOs reported that 77.7% were complying with the health and safety policy as depicted in Table 10 below. Most (84.6%) of the management reported to be compliant with the health and safety policy, with 7.7% accounting for non-compliant as well as uncertain for each.

**Table 10:** Compliance to Occupational Health and Safety requirements

HCWOs				Management		
**Legal requirements**	**Compliant (%)**	**Non- compliant (%)**	**Uncertain (%)**	**Compliant (%)**	**Non- compliant (%)**	**Uncertain (%)**
Health and safety policy	77.7	7.5	14.8	84.6	7.7	7.7
Appointment of Health and safety representa- tives	92.6	3.7	3.7	84.6	7.7	7.7
Training of health and safety representa- tives	81.5	7.4	11.1	92.3	7.7	0
Induction training of workers	92.6	3.7	3.7	84.6	7.7	7.7
Disaster/ Emergency plan	85.2	3.7	11.1	92.3	7.7	0
Conduction of Health risk assessment	85.2	3.7	11.1	76.9	15.4	7.7

The management of the HCFs were asked about their understanding regarding Section 16.2 appointment in terms of the Occupational Health and Safety Act 85 of 1993 [8]. A total of 62% of management were unclear of the requirement of Section 16.2 appointment. This was interesting as only 38% reported that it is the CEO who should ensure that health and safety system at the HCF is implemented.

### Integration of environmental pollution into health care waste management plans

Understanding of various environmental principles (green procurement, precautionary principle, polluter pays and duty of care) was asked to the HCWOs and the following results are depicted at Table 11. The green procurement was the most familiar environmental principle (59%), but the polluter pays principle and duty of care were the least known principles.

**Table 11:** Understanding of the environmental principles by health care waste officer

Environmental principles	Responses	Frequency (n)	Percentage (%)
The polluter pays principles	Environmental liability	10	29.4
	Unclear	24	70.6
Green procurement	Environmentally friendly products and services to avoid pollution	20	58.8
	Unclear	14	41.2
The precautionary principle	Action to prevent adverse or irreversible environmental and public health damage	6	17.6
	Unclear	28	82.4
Duty of care	Taking all the steps to ensure that waste is managed properly	10	29.0
	Hazardous waste tracking/ management from point of generation until its treatment and disposal	24	71.0

Management was asked on their responsibilities relating to environmental liabilities. Most (84.7%) of the management were unclear on what environmental liability entailed, with only 15.3% being clear on the principles. Only 53.8% of management of the HCFs were familiar with the concept of cradle to grave principle. Health care waste officers were asked about activities they perceived to be part of the cradle to grave principle. They indicated that generation at source (94.1%) is seen to be part of the cradle to grave.

**Fig. 2:**
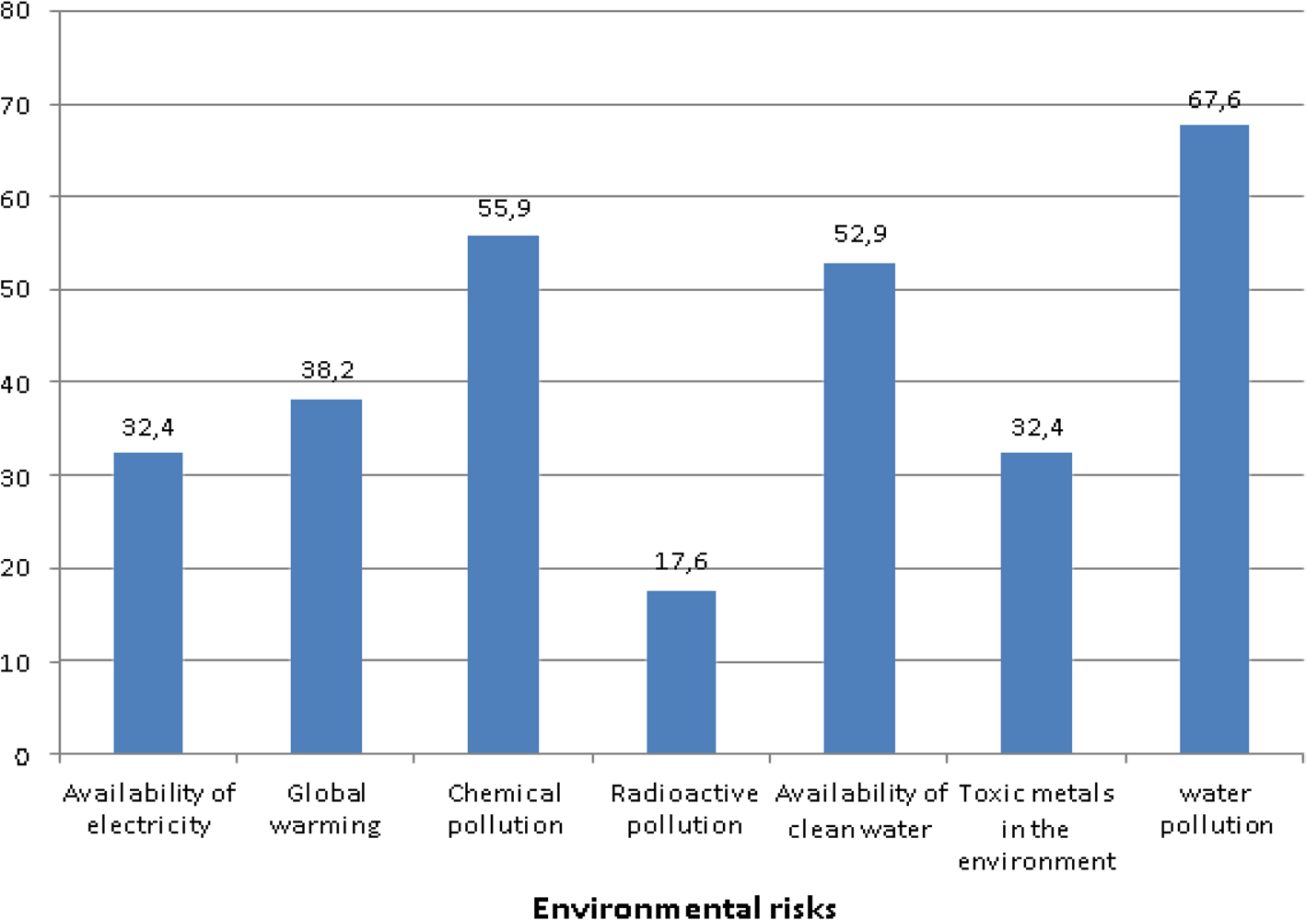
Environmental risks applicable to the health care facilities

The following question: “Indicate four environmental risks applicable to your HCFs”? was asked to the HCWOs. Figure 2 illustrates that water pollution (67.9%), followed by chemical pollution (55.9%) were the highest environmental risks identified.

**Fig. 3:**
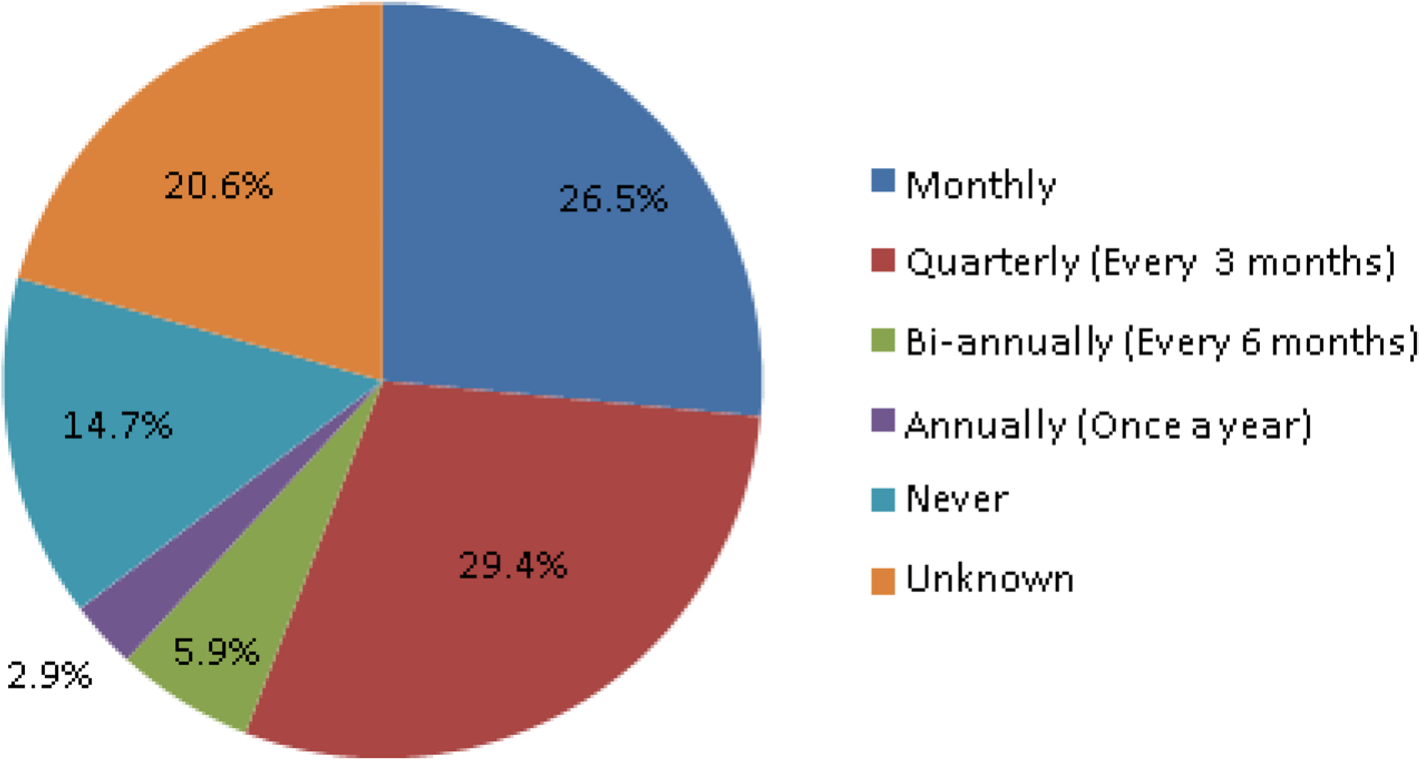
Frequency of water samples within the health care facilities

HCWO were asked how often they take water samples at their HCFs. Figure 3 below illustrates the frequencies of water samples taken with majority being on a quarterly basis (29.4%) at the HCF.

HCWO were asked if they had any formal conservation strategies at their HCFs. The questionnaire unravelled the importance of implementation of water and electricity saving strategies and HCW minimization and recycling initiatives, with only 14.7% of the HCFs having strategies in place to save water, electricity saving strategies (17.6%) and health care waste minimization strategies (55.9%). In essence, 70% of Health Care Facilities reported that they have implemented strategies on recycling paper, plastic, or cardboard. Table 12 shows the various means of conserving electricity, water, and decreasing of HCW and recycling of paper, plastic, or cardboard. Fewer strategies are in place, with the recycling of paper, plastic, being given highest priority.

**Table 12:** Formal environmental conservation strategies within health care facilities

	**Conservation strategies**	**Frequency (n)**	**Percentage (%)**
**Electricity saving**	Switching lights off at night or if not in use	2	5.8
	Computers and other appliance such as aircons are switched off	2	5.8
	Energy saving bulbs	2	5.8
	Use steam to heat water instead of geyser	1	2.9
**Water saving**	Fix all leaking taps/ plumbing systems timeously	4	11.8
	Close taps when not in use	1	2.9
	Training and education	9	26.4
**Health care waste minimisation**	Strict monitoring of segregation of waste/continuous audits	7	20.5
	Recycling	2	5.8
	Awareness campaigns	2	5.8
	EHP employed to monitor HCW	1	2.9
**Recycling of paper/ plastic/ cardboard**	Paper and cardboard is recycled on a continuous basis	10	29.4
	Service provider manages waste and collect on site	7	20.5
	Separated at the storage area	2	5.8
	Through community and informally	2	5.8

All the HCWOs reported having an HCRW service provider with the relevant air emission license for incineration. Majority (97%) are in possession of a hazardous waste transporter license, transfer facility license (94%) and 97% have proof of a waste treatment license in place.

### Integration of community participation into health care waste management plan

Table 13 depicts the type of HCW that is generated and being recycled within the HCF. Office paper was reported as the most recycled (44.1%) by the HCWOs, followed by newspaper/magazines.

**Table 13:** Recycling of waste types within health care waste facilities

Type of waste	Frequency (n)	Percentage (%)
Office Paper	15	44.1
Newspaper/magazines	4	11.8
Plastic	3	8.8
Aluminium Cans	3	8.8
Glass	1	2.9

HCWOs were asked how they manage HCRW issued by their HCF that is generated at home such as needles, syringes and test strips from a diabetic patient, unused or expired medication and dialysis bag from peritoneal dialysis patient. Issuing of small sharps containers for the diabetic patients was reported to be the highest (67.1%). They reported that patients are educated on the use and to return it when collecting their new medication. Unused or expired medication accounted for 32.3% and dialysis bags from peritoneal dialysis patients accounted for 2.9%.

**Fig. 4:**
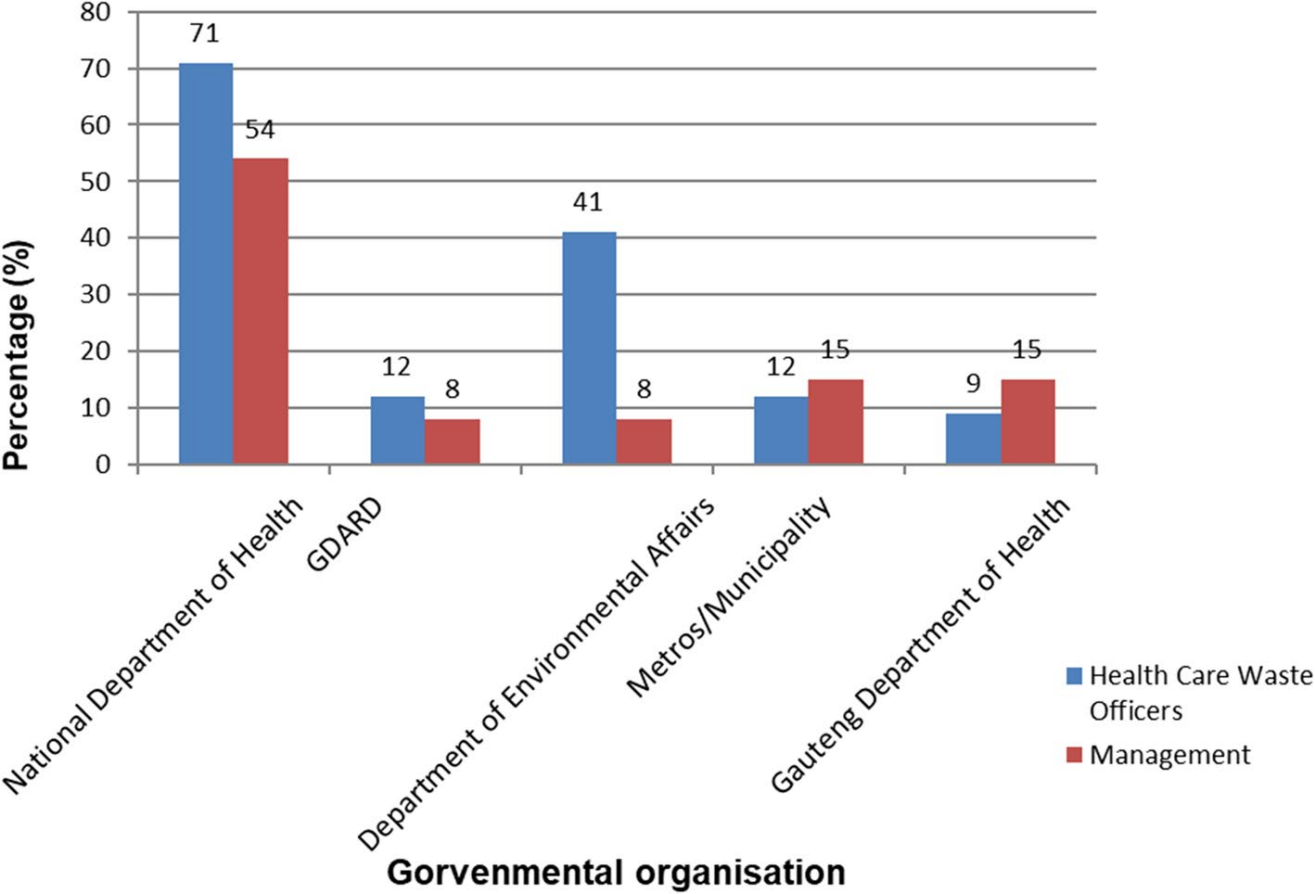
Governmental organisations responsible for the management of health care waste

The HCWO and management were asked about the governmental department to be the custodian of managing health care waste. HCWOs preferred the National Department of Health (70.6%) to be the custodian of HCW management within HCFs, and the provincial Gauteng Department of Health being the least preferred (8.8%). The management of HCFs also preferred the National Department of Health to be the custodian of HCW management within the HCFs, and the Department of Environmental and the Provincial Gauteng Department of Agriculture and Rural Development being the least preferred. Based on these findings, there was a fragmented approach in the management of health care waste: dealt with at both the National Department of Health, and the Department of Environment. Figure 4 below illustrate a comparison of the responses of the HCWOs and management on government department to be responsible for the management of health care waste.

## Discussion

This situational study is the first study to evaluate the status of HCWMPs in Gauteng Province, South Africa. Based on the findings, majority of HCWO had degrees and practicing as environmental health practitioners although in some facilities the cleaners were responsible for managing HCW. This can create a gap as cleaners will not be able to understand the management of HCW due to the educational background.

The HCWMPs were available in majority of HCFs. Both the HCWOs and management reported to not revert to the HCWMPs when decisions had to be made although they both believed the HCWMP is a necessary tool for management of HCW. The low usage reporting indicates that there is a gap with regards to the HCWMP.

Based on the study, appointment of HCW committees was very poor across the HCFs with limited professionals such as Pharmacy, skills development officials and procurement. Important legislative framework was also not used to guide the development of the HCWMPs such as Occupational Health and Safety Act of 1993 or National Waste strategy. Management was not clear on their role on health and safety matters relating to HCW and their liabilities as the 16.2 appointments in accordance with Occupational Health and Safety Act [8]. Environmental pollution is not addressed adequately within the HCFs as understanding amongst HCWOs and management on environmental principles was lacking. Subsequently, very few HCFs have environmental strategies in places such as electricity and water saving strategies. Global issues such as climate change and legislative aspects such as the polluter pays principle and the cradle to the grave concept are embedded in all environmental legislations. The change-over to renewable energy sources such as solar energy as well as saving of water and recycling of waste is also high on the agenda. HCFs are no exception and need to fully comply with environmental legislation.

Hangulu and Akintola [20] investigated management of HCW from community based care and found that there was poor HCW management practices among community workers because the policies from primary health services are not aligned with ensuring compliance. Although it was commendable that the Department of Health had managed to render health care at home, the HCRW generated is not taken into consideration. In this study, the integration of community participation into HCWMPs was not observed as HCFs observe minimum systems in place for managing HCRW that is generated at home. Health care waste such as needles, syringes, and test strips from diabetic patients, unused or expired medication, and dialysis bags from peritoneal dialysis patients generated from patients at home should also be formalised into such plans.

HCWO and management were requested to confirm which governmental department should be the custodian for managing HCW and the National Department of Health was the preferred custodian over the Department of Environmental Affairs which is the current custodian [20]. This means that synergy should be established or an agency responsible for health care waste management.

From the above mentioned, there is no integration of HCWMPs as inadequate health and safety aspects, environmental pollution as well community participation was reported upon.

According to the researcher`s knowledge, to date, this is the first study to be carried out in South Africa to evaluate the integration of HCWMPs. Qondile [12] and Mohamed et al. [14] looked at knowledge, attitude, and practices of health care waste management which was different from this study. The studies concluded that South Africa is facing a major challenge due to the unavailability of a national policy for health care risk waste management. A detailed HCWMP from cradle to grave is lacking on compliance to the set legislation [12, 14]. This study can therefore complement and add information to existing literature and help decision-makers or develop an efficient system.

## Conclusion

The aim of the study was to evaluate the integration of HCWMPs in public health care facilities in Gauteng province. The study therefore found that the current minimum requirements for HCWMPs are not fully integrated into occupational health and safety, environmental as well as community participation aspects.

Our findings confirmed the need to incorporate occupational health and safety matters such as training of staff, health risk assessments, employee, and employer responsibilities. Environmental principles should also be considered in the development of the HCWMP such as green procurement.

Regular awareness campaigns that involve the community should be done and matters such as management of HCRW at home should be advocated. Co-operation and integration within the different organisations and institutions such as non-governmental organisations and community leaders should be prioritised. Community health care workers should also be involved and included in the HCWMPs with reference to the management of HCW at facilities where they operate (practise) and to be the link between generation of HCW off-site and the respective HCFs.

The limitation of the study is that it focused on major generators. Although sufficient information was provided on the major generators, representative of other HCFs that generate less HCRW should be established.

## Supplementary Information

**Additional file 1:** **Table S1.** Options for variables identified in the study.

**Additional file 2:** **Table S2.** Variables and their description.

**Additional file 3. **

**Additional file 4.**

## Data Availability

All the data underlying the findings is contained within the manuscript.

## Abbreviations

CEO: Chief Executive Officer
EHP: Environmental Health Practitioner
HCF: Health care facilities
HCRW: Health care risk waste
HCW: Health care waste
HCWMPs: Health care waste management plans
HCWO: Health care waste officers
TUT: Tshwane University of Technology
