# Supplementary material for: Status of health care waste management plans and practices in public health care facilities in Gauteng Province, South Africa
Source: BMC Public Health. 2023 Feb 6;23:246. doi: 10.1186/s12889-023-15133-9 (PMC9901131; doi:10.1186/s12889-023-15133-9)
Supplement: Supplementary file 3 — Additional file 3. [file 12889_2023_15133_MOESM3_ESM.rtf]

Frequency Table


Interview Number	
	Frequency	Percent	Valid Percent	Cumulative Percent	
Valid	1	1	7.7	7.7	7.7	
	2	1	7.7	7.7	15.4	
	3	1	7.7	7.7	23.1	
	4	1	7.7	7.7	30.8	
	5	1	7.7	7.7	38.5	
	6	1	7.7	7.7	46.2	
	7	1	7.7	7.7	53.8	
	8	1	7.7	7.7	61.5	
	9	1	7.7	7.7	69.2	
	10	1	7.7	7.7	76.9	
	11	1	7.7	7.7	84.6	
	12	1	7.7	7.7	92.3	
	13	1	7.7	7.7	100.0	
	Total	13	100.0	100.0		


1. Education background	
	Frequency	Percent	Valid Percent	Cumulative Percent	
Valid	Medical Doctor	1	7.7	7.7	7.7	
	Health Sciences Post Degree	3	23.1	23.1	30.8	
	Health Sciences Degree	5	38.5	38.5	69.2	
	Other	4	30.8	30.8	100.0	
	Total	13	100.0	100.0		


1. Education background. Other (Specify)_________________	
	Frequency	Percent	Valid Percent	Cumulative Percent	
Valid		7	53.8	53.8	53.8	
	B-cur Nursing	1	7.7	7.7	61.5	
	B-Tech in nursing	1	7.7	7.7	69.2	
	Diploma in Nursing	1	7.7	7.7	76.9	
	Diploma in nUrsing B-Tech OHS	1	7.7	7.7	84.6	
	Diploma: Nursing, Degree: OHS and ISO 1400 and ISO 1800	1	7.7	7.7	92.3	
	MPH and MPA (Master's programme in Public and Development Management0	1	7.7	7.7	100.0	
	Total	13	100.0	100.0		


2. Have you received any HCW management training?	
	Frequency	Percent	Valid Percent	Cumulative Percent	
Valid	Yes	13	100.0	100.0	100.0	


3. If answered Yes to 2, please explain what kind of training was received?	
	Frequency	Percent	Valid Percent	Cumulative Percent	
Valid	4 CPD points training in HCW management	1	7.7	7.7	7.7	
	4 hour training managers	2	15.4	15.4	23.1	
	All the trainings, which include handling and the management of HCW in broad	1	7.7	7.7	30.8	
	All training, managemnt and lower level training e.g the handling of HCW	1	7.7	7.7	38.5	
	Categories of HCW, How to manage HCW in a facility and eeverything that has to do with its hazard and the OHS	1	7.7	7.7	46.2	
	Formal 4 hour training	1	7.7	7.7	53.8	
	HCW management training	1	7.7	7.7	61.5	
	Importance of HCW, waste segregation from managemnt, what is is and the effects and hazards	1	7.7	7.7	69.2	
	Management of HCW	2	15.4	15.4	84.6	
	Spectrum of managemnt of HCW	1	7.7	7.7	92.3	
	Training by Geo-zone	1	7.7	7.7	100.0	
	Total	13	100.0	100.0		


4. Are you familiar with Gauteng HCW Management Regulations, 2004?	
	Frequency	Percent	Valid Percent	Cumulative Percent	
Valid	Yes	11	84.6	84.6	84.6	
	No	2	15.4	15.4	100.0	
	Total	13	100.0	100.0		


5. Is management of HCW included in your job description?	
	Frequency	Percent	Valid Percent	Cumulative Percent	
Valid	Yes	7	53.8	53.8	53.8	
	No	6	46.2	46.2	100.0	
	Total	13	100.0	100.0		


6. How much HCW does your HCF produce in a day?	
	Frequency	Percent	Valid Percent	Cumulative Percent	
Valid	11 to 20 kg	3	23.1	23.1	23.1	
	More than 20kg	10	76.9	76.9	100.0	
	Total	13	100.0	100.0		


7. Does your HCF have a Health Care Waste Management Plan (HCWMP) in place?	
	Frequency	Percent	Valid Percent	Cumulative Percent	
Valid	Yes	11	84.6	84.6	84.6	
	No	2	15.4	15.4	100.0	
	Total	13	100.0	100.0		


8. What is your role in the implementation of HCWMP?	
	Frequency	Percent	Valid Percent	Cumulative Percent	
Valid		1	7.7	7.7	7.7	
	As a manager, I have a role to oversee everything	1	7.7	7.7	15.4	
	Developed the plan	1	7.7	7.7	23.1	
	development of the HCW plan to ensure proper segreagtion and recycling of general waste	1	7.7	7.7	30.8	
	Ensure it is executed, hospital is hazard free	1	7.7	7.7	38.5	
	Ensure that all is in order	1	7.7	7.7	46.2	
	Ensure that consumables are available and training is conducted	1	7.7	7.7	53.8	
	Ensure that is completed and submitted to the relevant authorities	1	7.7	7.7	61.5	
	ensure that the plan is developed	1	7.7	7.7	69.2	
	Facilitated the process of ensuring that it is implemented and approval was granted	1	7.7	7.7	76.9	
	Implementation	1	7.7	7.7	84.6	
	management role	1	7.7	7.7	92.3	
	Oversee that it is developed and approved	1	7.7	7.7	100.0	
	Total	13	100.0	100.0		


9. Do you think the current HCWMP is a necessary tool that promotes the management of HCW in your facility?	
	Frequency	Percent	Valid Percent	Cumulative Percent	
Valid	Yes	13	100.0	100.0	100.0	


10. How often do you refer back to the HCWMP when strategic decisions have to be made regarding HCW?	
	Frequency	Percent	Valid Percent	Cumulative Percent	
Valid	Always	7	53.8	53.8	53.8	
	Occasionally	6	46.2	46.2	100.0	
	Total	13	100.0	100.0		


11. Is there a dedicated person appointed to manage HCW at your (Health Care Facility) HCF?	
	Frequency	Percent	Valid Percent	Cumulative Percent	
Valid	Yes	10	76.9	76.9	76.9	
	No	3	23.1	23.1	100.0	
	Total	13	100.0	100.0		


12. If answered Yes to question 11, what is the job tile of such person:	
	Frequency	Percent	Valid Percent	Cumulative Percent	
Valid		2	15.4	15.4	15.4	
	EHP	6	46.2	46.2	61.5	
	Nurse and EH and student EHP	1	7.7	7.7	69.2	
	Nursing: Infection prevention and control	1	7.7	7.7	76.9	
	OHS co-ordinator and EHP	1	7.7	7.7	84.6	
	Prof. Nurse	1	7.7	7.7	92.3	
	Prof. Nurse and cleaner	1	7.7	7.7	100.0	
	Total	13	100.0	100.0		


13. If answered No to 11, what is the reason?	
	Frequency	Percent	Valid Percent	Cumulative Percent	
Valid		10	76.9	76.9	76.9	
	1	1	7.7	7.7	84.6	
	A team is managing HCW	1	7.7	7.7	92.3	
	I am currently the Acting manager	1	7.7	7.7	100.0	
	Total	13	100.0	100.0		


14. Has an Integrated HCW committee been appointed at your HCF?	
	Frequency	Percent	Valid Percent	Cumulative Percent	
Valid	Yes	4	30.8	33.3	33.3	
	No	8	61.5	66.7	100.0	
	Total	12	92.3	100.0		
Missing	System	1	7.7			
Total	13	100.0			


15. Please give detail to which professions does the integrated HCW committee members belongs? Member 1	
	Frequency	Percent	Valid Percent	Cumulative Percent	
Valid		8	61.5	61.5	61.5	
	General workers	1	7.7	7.7	69.2	
	Nurse	1	7.7	7.7	76.9	
	Nursing manager	1	7.7	7.7	84.6	
	OHS  co-ordinator	1	7.7	7.7	92.3	
	Support- Security/ cleaners	1	7.7	7.7	100.0	
	Total	13	100.0	100.0		


15. Please give detail to which professions does the integrated HCW committee members belongs? Member 2	
	Frequency	Percent	Valid Percent	Cumulative Percent	
Valid		8	61.5	61.5	61.5	
	Admin/ clerks	1	7.7	7.7	69.2	
	EHP	1	7.7	7.7	76.9	
	Nursing manager	1	7.7	7.7	84.6	
	Pharmacy	1	7.7	7.7	92.3	
	Waste collectors	1	7.7	7.7	100.0	
	Total	13	100.0	100.0		


15. Please give detail to which professions does the integrated HCW committee members belongs? Member 3	
	Frequency	Percent	Valid Percent	Cumulative Percent	
Valid		8	61.5	61.5	61.5	
	EHP	1	7.7	7.7	69.2	
	Nursing	1	7.7	7.7	76.9	
	Pharmacy	1	7.7	7.7	84.6	
	Pharmacy manager	1	7.7	7.7	92.3	
	Procurement	1	7.7	7.7	100.0	
	Total	13	100.0	100.0		


15. Please give detail to which professions does the integrated HCW committee members belongs? Member 4	
	Frequency	Percent	Valid Percent	Cumulative Percent	
Valid		8	61.5	61.5	61.5	
	Doctors	1	7.7	7.7	69.2	
	Finance	1	7.7	7.7	76.9	
	Infection Prevention and control	1	7.7	7.7	84.6	
	Nursing manager	1	7.7	7.7	92.3	
	Pharmacy	1	7.7	7.7	100.0	
	Total	13	100.0	100.0		


15. Please give detail to which professions does the integrated HCW committee members belongs? Member 5	
	Frequency	Percent	Valid Percent	Cumulative Percent	
Valid		8	61.5	61.5	61.5	
	Alied workers	1	7.7	7.7	69.2	
	Doctors	1	7.7	7.7	76.9	
	OHS nurse	1	7.7	7.7	84.6	
	Procurement	1	7.7	7.7	92.3	
	supply chain	1	7.7	7.7	100.0	
	Total	13	100.0	100.0		


15. Please give detail to which professions does the integrated HCW committee members belongs? Member 6	
	Frequency	Percent	Valid Percent	Cumulative Percent	
Valid		10	76.9	76.9	76.9	
	Clinical manager	1	7.7	7.7	84.6	
	EHP	1	7.7	7.7	92.3	
	HCW collectors	1	7.7	7.7	100.0	
	Total	13	100.0	100.0		


15. Please give detail to which professions does the integrated HCW committee members belongs? Member 7	
	Frequency	Percent	Valid Percent	Cumulative Percent	
Valid		11	84.6	84.6	84.6	
	cleaning services	1	7.7	7.7	92.3	
	Quality assurance	1	7.7	7.7	100.0	
	Total	13	100.0	100.0		


15. Please give detail to which professions does the integrated HCW committee members belongs? Member 8	
	Frequency	Percent	
Missing	System	13	100.0	


16. How often does the HCW committee meet?	
	Frequency	Percent	Valid Percent	Cumulative Percent	
Valid	Other (specify)	5	38.5	100.0	100.0	
Missing	System	8	61.5			
Total	13	100.0			


16. How often does the HCW committee meet? Other (specify)  __________________	
	Frequency	Percent	Valid Percent	Cumulative Percent	
Valid		8	61.5	61.5	61.5	
	Every quarter	1	7.7	7.7	69.2	
	Monthly	3	23.1	23.1	92.3	
	Quarter	1	7.7	7.7	100.0	
	Total	13	100.0	100.0		


17. Do the job descriptions of the Integrated HCW committee members include the management of HCW? Member 1	
	Frequency	Percent	Valid Percent	Cumulative Percent	
Valid	Yes	5	38.5	100.0	100.0	
Missing	System	8	61.5			
Total	13	100.0			


17. Do the job descriptions of the Integrated HCW committee members include the management of HCW? Member 1	
	Frequency	Percent	Valid Percent	Cumulative Percent	
Valid	Yes	4	30.8	80.0	80.0	
	Uncertain	1	7.7	20.0	100.0	
	Total	5	38.5	100.0		
Missing	System	8	61.5			
Total	13	100.0			


17. Do the job descriptions of the Integrated HCW committee members include the management of HCW? Member 1	
	Frequency	Percent	Valid Percent	Cumulative Percent	
Valid	Yes	1	7.7	20.0	20.0	
	No	1	7.7	20.0	40.0	
	Uncertain	3	23.1	60.0	100.0	
	Total	5	38.5	100.0		
Missing	System	8	61.5			
Total	13	100.0			


17. Do the job descriptions of the Integrated HCW committee members include the management of HCW? Member 1	
	Frequency	Percent	Valid Percent	Cumulative Percent	
Valid	Yes	2	15.4	40.0	40.0	
	No	2	15.4	40.0	80.0	
	Uncertain	1	7.7	20.0	100.0	
	Total	5	38.5	100.0		
Missing	System	8	61.5			
Total	13	100.0			


17. Do the job descriptions of the Integrated HCW committee members include the management of HCW? Member 1	
	Frequency	Percent	Valid Percent	Cumulative Percent	
Valid	Yes	2	15.4	40.0	40.0	
	No	1	7.7	20.0	60.0	
	Uncertain	2	15.4	40.0	100.0	
	Total	5	38.5	100.0		
Missing	System	8	61.5			
Total	13	100.0			


17. Do the job descriptions of the Integrated HCW committee members include the management of HCW? Member 1	
	Frequency	Percent	Valid Percent	Cumulative Percent	
Valid	Yes	2	15.4	66.7	66.7	
	No	1	7.7	33.3	100.0	
	Total	3	23.1	100.0		
Missing	System	10	76.9			
Total	13	100.0			


17. Do the job descriptions of the Integrated HCW committee members include the management of HCW? Member 1	
	Frequency	Percent	Valid Percent	Cumulative Percent	
Valid	Yes	2	15.4	100.0	100.0	
Missing	System	11	84.6			
Total	13	100.0			


17. Do the job descriptions of the Integrated HCW committee members include the management of HCW? Member 1	
	Frequency	Percent	
Missing	System	13	100.0	


18. If answered No to 17, what is the reason? Member 1	
	Frequency	Percent	Valid Percent	Cumulative Percent	
Valid		13	100.0	100.0	100.0	


18. If answered No to 17, what is the reason? Member 2	
	Frequency	Percent	Valid Percent	Cumulative Percent	
Valid		12	92.3	92.3	92.3	
	Not part of their scope	1	7.7	7.7	100.0	
	Total	13	100.0	100.0		


18. If answered No to 17, what is the reason? Member 3	
	Frequency	Percent	Valid Percent	Cumulative Percent	
Valid		12	92.3	92.3	92.3	
	Not part of their KPA	1	7.7	7.7	100.0	
	Total	13	100.0	100.0		


18. If answered No to 17, what is the reason? Member 4	
	Frequency	Percent	Valid Percent	Cumulative Percent	
Valid		11	84.6	84.6	84.6	
	Clinical manager is responsible- Lac of doctors	1	7.7	7.7	92.3	
	Not part of their KPA	1	7.7	7.7	100.0	
	Total	13	100.0	100.0		


18. If answered No to 17, what is the reason? Member 5	
	Frequency	Percent	Valid Percent	Cumulative Percent	
Valid		12	92.3	92.3	92.3	
	Scope too broad	1	7.7	7.7	100.0	
	Total	13	100.0	100.0		


18. If answered No to 17, what is the reason? Member 6	
	Frequency	Percent	
Missing	System	13	100.0	


18. If answered No to 17, what is the reason? Member 7	
	Frequency	Percent	
Missing	System	13	100.0	


18. If answered No to 17, what is the reason? Member 8	
	Frequency	Percent	
Missing	System	13	100.0	


19. Please indicate the extent to which the following statements listed below are considered a priority at your HCF? Conducting inspections	
	Frequency	Percent	Valid Percent	Cumulative Percent	
Valid	Higher priority	13	100.0	100.0	100.0	


19. Please indicate the extent to which the following statements listed below are considered a priority at your HCF? Feedback regarding inspection findings	
	Frequency	Percent	Valid Percent	Cumulative Percent	
Valid	Higher priority	13	100.0	100.0	100.0	


19. Please indicate the extent to which the following statements listed below are considered a priority at your HCF? Education and providing information	
	Frequency	Percent	Valid Percent	Cumulative Percent	
Valid	Higher priority	13	100.0	100.0	100.0	


20. What are your responsibilities regarding the following legal requirements which are applicable to HCW? Environmental liability	
	Frequency	Percent	Valid Percent	Cumulative Percent	
Valid		6	46.2	46.2	46.2	
	Environment is safe should there be e.g. chemicals that the CEO will be personally liable.	1	7.7	7.7	53.8	
	Eveyone is liable to take care of the environment	1	7.7	7.7	61.5	
	Management of waste to avoid any spillages	1	7.7	7.7	69.2	
	Pollution affects other people from the community	1	7.7	7.7	76.9	
	The CEO is liable for any pollution	1	7.7	7.7	84.6	
	The hospital should account when the environmnet is polluted	1	7.7	7.7	92.3	
	Those who pollute must be resposible for cost of damage	1	7.7	7.7	100.0	
	Total	13	100.0	100.0		


20. What are your responsibilities regarding the following legal requirements which are applicable to HCW? Cradle to grave	
	Frequency	Percent	Valid Percent	Cumulative Percent	
Valid		3	23.1	23.1	23.1	
	A standard that ensures that waste managemnt takes place.	1	7.7	7.7	30.8	
	Environment should be cared for	1	7.7	7.7	38.5	
	From generation till treatment	1	7.7	7.7	46.2	
	Manangement of HCW waste from the point of generation until the treatment and disposal	1	7.7	7.7	53.8	
	MAnangement of HCW waste from the point of generation until the treatment and disposal	1	7.7	7.7	61.5	
	Taking care of the waste from point of generation until disposal	1	7.7	7.7	69.2	
	The  hospital  should take responsibility to ensure that waste is destroyed in accordance.	1	7.7	7.7	76.9	
	The management of waste until the final stage of disposal	1	7.7	7.7	84.6	
	The word is commonly used but meaning is unknown	1	7.7	7.7	92.3	
	What happens to our waste	1	7.7	7.7	100.0	
	Total	13	100.0	100.0		


20. What are your responsibilities regarding the following legal requirements which are applicable to HCW? 16.2 appointment	
	Frequency	Percent	Valid Percent	Cumulative Percent	
Valid		7	53.8	53.8	53.8	
	Appointmnet of the HCW officer in writing and signed by the CEO	1	7.7	7.7	61.5	
	CEO	2	15.4	15.4	76.9	
	CEO appointment according to OHS Act	1	7.7	7.7	84.6	
	Health & safety	1	7.7	7.7	92.3	
	The manager	1	7.7	7.7	100.0	
	Total	13	100.0	100.0		


21. Is there a system that ensures retrievability and accessibility of HCW documentation?	
	Frequency	Percent	Valid Percent	Cumulative Percent	
Valid	Yes	13	100.0	100.0	100.0	


22. If answered Yes to 21, please explain the system that ensures retrievability and accessibility of HCW documentation?	
	Frequency	Percent	Valid Percent	Cumulative Percent	
Valid		2	15.4	15.4	15.4	
	A filling system	1	7.7	7.7	23.1	
	All copies are e-copied	1	7.7	7.7	30.8	
	Copies are made for logistics manager and waste collectors	1	7.7	7.7	38.5	
	Files are accessible to all staff	1	7.7	7.7	46.2	
	key stakeholders have e-copies and the master file is available at the file cabinet for all to acess	1	7.7	7.7	53.8	
	Labelled, index files and documents displayed on the board	1	7.7	7.7	61.5	
	Manual hard copies	1	7.7	7.7	69.2	
	Saved in accoradnce with the index	1	7.7	7.7	76.9	
	The documents are saved at reception	1	7.7	7.7	84.6	
	There are more than one person working with HCW, so acess to documents is always gurantteed	1	7.7	7.7	92.3	
	We file all documents	1	7.7	7.7	100.0	
	Total	13	100.0	100.0		


23. Is there a system that is used to determine the training needs for officials (continuous development) on HCW?	
	Frequency	Percent	Valid Percent	Cumulative Percent	
Valid	Yes	13	100.0	100.0	100.0	


24. Is training of HCW taking place in your HCF?	
	Frequency	Percent	Valid Percent	Cumulative Percent	
Valid	Yes	13	100.0	100.0	100.0	


25. If answered Yes to 24, indicate what kind of training is given?	
	Frequency	Percent	Valid Percent	Cumulative Percent	
Valid	Paid consultants	12	92.3	100.0	100.0	
Missing	System	1	7.7			
Total	13	100.0			


26. If answered No or Uncertain at 24; indicate which of the following reasons prevents your facility from providing training?	
	Frequency	Percent	
Missing	System	13	100.0	


26. If answered No or Uncertain at 24; indicate which of the following reasons prevents your facility from providing training? Other(specify)_________________	
	Frequency	Percent	Valid Percent	Cumulative Percent	
Valid	1	1	7.7	100.0	100.0	
Missing	System	12	92.3			
Total	13	100.0			


27. How does your HCF address HCW shortfalls that may need budget?	
	Frequency	Percent	Valid Percent	Cumulative Percent	
Valid		1	7.7	7.7	7.7	
	Annual plan	1	7.7	7.7	15.4	
	Annually a financial plan is submitted with all the items listed that will be needed on that year.	1	7.7	7.7	23.1	
	By means of motivations	1	7.7	7.7	30.8	
	CEO is notified of the needs and if approved, funds are made available	1	7.7	7.7	38.5	
	files are done	1	7.7	7.7	46.2	
	management is notified by mens of a motivation	1	7.7	7.7	53.8	
	Motivation and submission	1	7.7	7.7	61.5	
	Submission of a motivation	1	7.7	7.7	69.2	
	Supply sent for proposal and itemized	1	7.7	7.7	76.9	
	Through the financial manager	1	7.7	7.7	84.6	
	Through the system of motivations	1	7.7	7.7	92.3	
	Very difficult	1	7.7	7.7	100.0	
	Total	13	100.0	100.0		


28. Indicate if the following aspects are complied with regarding Health Care Risk Waste (HCRW) management at your HCF? Health and safety policy	
	Frequency	Percent	Valid Percent	Cumulative Percent	
Valid	Yes	11	84.6	84.6	84.6	
	No	1	7.7	7.7	92.3	
	Uncertain	1	7.7	7.7	100.0	
	Total	13	100.0	100.0		


28. Indicate if the following aspects are complied with regarding Health Care Risk Waste (HCRW) management at your HCF? Appointment of Health and safety representatives	
	Frequency	Percent	Valid Percent	Cumulative Percent	
Valid	Yes	11	84.6	84.6	84.6	
	No	1	7.7	7.7	92.3	
	Uncertain	1	7.7	7.7	100.0	
	Total	13	100.0	100.0		


28. Indicate if the following aspects are complied with regarding Health Care Risk Waste (HCRW) management at your HCF? Training of health and safety representatives	
	Frequency	Percent	Valid Percent	Cumulative Percent	
Valid	Yes	12	92.3	92.3	92.3	
	Uncertain	1	7.7	7.7	100.0	
	Total	13	100.0	100.0		


28. Indicate if the following aspects are complied with regarding Health Care Risk Waste (HCRW) management at your HCF? Induction training of workers	
	Frequency	Percent	Valid Percent	Cumulative Percent	
Valid	Yes	11	84.6	84.6	84.6	
	No	2	15.4	15.4	100.0	
	Total	13	100.0	100.0		


28. Indicate if the following aspects are complied with regarding Health Care Risk Waste (HCRW) management at your HCF? Disaster/ Emergency plan	
	Frequency	Percent	Valid Percent	Cumulative Percent	
Valid	Yes	12	92.3	92.3	92.3	
	No	1	7.7	7.7	100.0	
	Total	13	100.0	100.0		


28. Indicate if the following aspects are complied with regarding Health Care Risk Waste (HCRW) management at your HCF? Health risk assessments conducted	
	Frequency	Percent	Valid Percent	Cumulative Percent	
Valid	Yes	10	76.9	76.9	76.9	
	No	2	15.4	15.4	92.3	
	Uncertain	1	7.7	7.7	100.0	
	Total	13	100.0	100.0		


29. In your opinion, which governmental organisation is responsible to ensure HCW is managed within the HCF?	
	Frequency	Percent	Valid Percent	Cumulative Percent	
Valid	National Department of Health	7	53.8	53.8	53.8	
	Gauteng Department of Agriculture and Rural Development	1	7.7	7.7	61.5	
	Department of Environmental Affairs	1	7.7	7.7	69.2	
	Municipality/ Metro	2	15.4	15.4	84.6	
	Gauteng Department of Health	2	15.4	15.4	100.0	
	Total	13	100.0	100.0		
