# Supplementary material for: Status of health care waste management plans and practices in public health care facilities in Gauteng Province, South Africa
Source: BMC Public Health. 2023 Feb 6;23:246. doi: 10.1186/s12889-023-15133-9 (PMC9901131; doi:10.1186/s12889-023-15133-9)
Supplement: Supplementary file 4 — Additional file 4. [file 12889_2023_15133_MOESM4_ESM.rtf]

Frequency Table


Questionnaire	
	Frequency	Percent	Valid Percent	Cumulative Percent	
Valid	1	1	2.9	2.9	2.9	
	2	1	2.9	2.9	5.9	
	3	1	2.9	2.9	8.8	
	4	1	2.9	2.9	11.8	
	5	1	2.9	2.9	14.7	
	6	1	2.9	2.9	17.6	
	7	1	2.9	2.9	20.6	
	8	1	2.9	2.9	23.5	
	9	1	2.9	2.9	26.5	
	10	1	2.9	2.9	29.4	
	11	1	2.9	2.9	32.4	
	12	1	2.9	2.9	35.3	
	13	1	2.9	2.9	38.2	
	14	1	2.9	2.9	41.2	
	15	1	2.9	2.9	44.1	
	16	1	2.9	2.9	47.1	
	17	1	2.9	2.9	50.0	
	18	1	2.9	2.9	52.9	
	19	1	2.9	2.9	55.9	
	20	1	2.9	2.9	58.8	
	21	1	2.9	2.9	61.8	
	22	1	2.9	2.9	64.7	
	23	1	2.9	2.9	67.6	
	24	1	2.9	2.9	70.6	
	25	1	2.9	2.9	73.5	
	26	1	2.9	2.9	76.5	
	27	1	2.9	2.9	79.4	
	28	1	2.9	2.9	82.4	
	29	1	2.9	2.9	85.3	
	30	1	2.9	2.9	88.2	
	31	1	2.9	2.9	91.2	
	32	1	2.9	2.9	94.1	
	33	1	2.9	2.9	97.1	
	34	1	2.9	2.9	100.0	
	Total	34	100.0	100.0		


1.1 Gender	
	Frequency	Percent	Valid Percent	Cumulative Percent	
Valid	Male	6	17.6	17.6	17.6	
	Female	28	82.4	82.4	100.0	
	Total	34	100.0	100.0		


1.2. Highest level of education completed?	
	Frequency	Percent	Valid Percent	Cumulative Percent	
Valid	Matric	2	5.9	5.9	5.9	
	Post Matric Diploma	11	32.4	32.4	38.2	
	Post Matric Degree	16	47.1	47.1	85.3	
	Postgraduate	3	8.8	8.8	94.1	
	Certificate	2	5.9	5.9	100.0	
	Total	34	100.0	100.0		


1.3. What is your position in the HCF (job title)?	
	Frequency	Percent	Valid Percent	Cumulative Percent	
Valid	Environmental Health Practitioner	19	55.9	55.9	55.9	
	Infection Control Nurse	8	23.5	23.5	79.4	
	Health Care Waste Officer	3	8.8	8.8	88.2	
	Nursing Manager	2	5.9	5.9	94.1	
	Other (specify) _________________	2	5.9	5.9	100.0	
	Total	34	100.0	100.0		


1.3. What is your position in the HCF (job title)? Other (specify) _________________	
	Frequency	Percent	Valid Percent	Cumulative Percent	
Valid		32	94.1	94.1	94.1	
	health care waste officer/infection  prevention and control/ OHS Nurse	1	2.9	2.9	97.1	
	Procurement Officer	1	2.9	2.9	100.0	
	Total	34	100.0	100.0		


1.4. In which district is your HCF?	
	Frequency	Percent	Valid Percent	Cumulative Percent	
Valid	Ekurhuleni	7	20.6	20.6	20.6	
	City of Joburg	10	29.4	29.4	50.0	
	Sedibeng	4	11.8	11.8	61.8	
	City of Tshwane	10	29.4	29.4	91.2	
	West Rand	3	8.8	8.8	100.0	
	Total	34	100.0	100.0		


1.5. Indicate the type of your HCF?	
	Frequency	Percent	Valid Percent	Cumulative Percent	
Valid	Tertiary Hospital	3	8.8	8.8	8.8	
	Regional Hospital	10	29.4	29.4	38.2	
	District Hospital	12	35.3	35.3	73.5	
	Community Health Centre	5	14.7	14.7	88.2	
	Maternity and obstetrics Unit	1	2.9	2.9	91.2	
	Academic Hospital	3	8.8	8.8	100.0	
	Total	34	100.0	100.0		


1.6. Have you received training about Health Care Waste (HCW) Management?	
	Frequency	Percent	Valid Percent	Cumulative Percent	
Valid	Yes	33	97.1	97.1	97.1	
	No	1	2.9	2.9	100.0	
	Total	34	100.0	100.0		


1.7. Are you familiar with Gauteng HCW Management Regulations, 2004?	
	Frequency	Percent	Valid Percent	Cumulative Percent	
Valid	Yes	34	100.0	100.0	100.0	


2.1. What type of HCW does your HCF generate	
	Frequency	Percent	Valid Percent	Cumulative Percent	
Valid	1,2,3,4,5,6,7	1	2.9	2.9	2.9	
	1,2,3,4,5,6,7,8	5	14.7	14.7	17.6	
	1,2,3,4,5,6,8	1	2.9	2.9	20.6	
	1,2,34,5,6,7	1	2.9	2.9	23.5	
	1,2,4,5,6,7	1	2.9	2.9	26.5	
	1,2,4,5,6,7,8	4	11.8	11.8	38.2	
	1,2,4,5,7	6	17.6	17.6	55.9	
	1,2,4,5,7,8	12	35.3	35.3	91.2	
	1,2,4,6	1	2.9	2.9	94.1	
	1,2,4,7	2	5.9	5.9	100.0	
	Total	34	100.0	100.0		


2.1. What type of HCW does your HCF generate...Pathological	
	Frequency	Percent	Valid Percent	Cumulative Percent	
Valid	Pathological	34	100.0	100.0	100.0	


2.1. What type of HCW does your HCF generate...Sharps	
	Frequency	Percent	Valid Percent	Cumulative Percent	
Valid	Sharps	34	100.0	100.0	100.0	


2.1. What type of HCW does your HCF generate...Cytotoxic waste	
	Frequency	Percent	Valid Percent	Cumulative Percent	
Valid	Cytotoxic waste	8	23.5	100.0	100.0	
Missing	System	26	76.5			
Total	34	100.0			


2.1. What type of HCW does your HCF generate...Pharamceutical waste	
	Frequency	Percent	Valid Percent	Cumulative Percent	
Valid	Pharamceutical waste	34	100.0	100.0	100.0	


2.1. What type of HCW does your HCF generate...Infectious waste	
	Frequency	Percent	Valid Percent	Cumulative Percent	
Valid	Infectious waste	31	91.2	100.0	100.0	
Missing	System	3	8.8			
Total	34	100.0			


2.1. What type of HCW does your HCF generate...Radioactive waste	
	Frequency	Percent	Valid Percent	Cumulative Percent	
Valid	Radioactive waste	14	41.2	100.0	100.0	
Missing	System	20	58.8			
Total	34	100.0			


2.1. What type of HCW does your HCF generate...General waste	
	Frequency	Percent	Valid Percent	Cumulative Percent	
Valid	General waste	32	94.1	100.0	100.0	
Missing	System	2	5.9			
Total	34	100.0			


2.1. What type of HCW does your HCF generate...Chemical waste	
	Frequency	Percent	Valid Percent	Cumulative Percent	
Valid	Chemical waste	22	64.7	100.0	100.0	
Missing	System	12	35.3			
Total	34	100.0			


2.2. In your opinion who ultimately takes responsibility for HCW at your HCF?	
	Frequency	Percent	Valid Percent	Cumulative Percent	
Valid	1	10	29.4	29.4	29.4	
	1,2,3	5	14.7	14.7	44.1	
	1,2,3,4	1	2.9	2.9	47.1	
	1,2,3,4,5	1	2.9	2.9	50.0	
	1,2,4	1	2.9	2.9	52.9	
	1,3	2	5.9	5.9	58.8	
	1.3	3	8.8	8.8	67.6	
	2	2	5.9	5.9	73.5	
	3	3	8.8	8.8	82.4	
	5	4	11.8	11.8	94.1	
	6	2	5.9	5.9	100.0	
	Total	34	100.0	100.0		


2.2. In your opinion who ultimately takes responsibility for HCW at your HCF? Environmental Health Practitioner	
	Frequency	Percent	Valid Percent	Cumulative Percent	
Valid	Environmental Health Practitioner	23	67.6	100.0	100.0	
Missing	System	11	32.4			
Total	34	100.0			


2.2. In your opinion who ultimately takes responsibility for HCW at your HCF? In Control Nurse	
	Frequency	Percent	Valid Percent	Cumulative Percent	
Valid	In Control Nurse	10	29.4	100.0	100.0	
Missing	System	24	70.6			
Total	34	100.0			


2.2. In your opinion who ultimately takes responsibility for HCW at your HCF? HCW Officer	
	Frequency	Percent	Valid Percent	Cumulative Percent	
Valid	HCW Officer	15	44.1	100.0	100.0	
Missing	System	19	55.9			
Total	34	100.0			


2.2. In your opinion who ultimately takes responsibility for HCW at your HCF? OHS Nurse	
	Frequency	Percent	Valid Percent	Cumulative Percent	
Valid	OHS Nurse	3	8.8	100.0	100.0	
Missing	System	31	91.2			
Total	34	100.0			


2.2. In your opinion who ultimately takes responsibility for HCW at your HCF? Nursing Manager	
	Frequency	Percent	Valid Percent	Cumulative Percent	
Valid	Nursing Manager	5	14.7	100.0	100.0	
Missing	System	29	85.3			
Total	34	100.0			


2.2. In your opinion who ultimately takes responsibility for HCW at your HCF? Other	
	Frequency	Percent	Valid Percent	Cumulative Percent	
Valid	Other	2	5.9	100.0	100.0	
Missing	System	32	94.1			
Total	34	100.0			


2.3. How much HCW does your HCF produce in a day?	
	Frequency	Percent	Valid Percent	Cumulative Percent	
Valid	11 to 20 kg	7	20.6	21.2	21.2	
	More than 20kg	26	76.5	78.8	100.0	
	Total	33	97.1	100.0		
Missing	System	1	2.9			
Total	34	100.0			


2.4. Does your HCF have a HCWMP in place?	
	Frequency	Percent	Valid Percent	Cumulative Percent	
Valid	Yes	27	79.4	79.4	79.4	
	No	7	20.6	20.6	100.0	
	Total	34	100.0	100.0		


2.5. How often do you revert back to HCWMP when operational decisions have to be made regarding HCW?	
	Frequency	Percent	Valid Percent	Cumulative Percent	
Valid	Always	6	17.6	22.2	22.2	
	Occasionally	20	58.8	74.1	96.3	
	Never	1	2.9	3.7	100.0	
	Total	27	79.4	100.0		
Missing	System	7	20.6			
Total	34	100.0			


2.6. Do you think the current HCWMP is a necessary tool that promotes the management of HCW in your HCF?	
	Frequency	Percent	Valid Percent	Cumulative Percent	
Valid	Yes	24	70.6	88.9	88.9	
	No	3	8.8	11.1	100.0	
	Total	27	79.4	100.0		
Missing	System	7	20.6			
Total	34	100.0			


2.7. Name the guiding document/s used as referrals to develop the HCWMP.	
	Frequency	Percent	Valid Percent	Cumulative Percent	
Valid		9	26.5	26.5	26.5	
	Code of practice for health care waste,The national environmental managemnt act No.107 of 1998	1	2.9	2.9	29.4	
	Code of practice for health care waste,The national environmental managemnt waste asct No.59 of 2008	1	2.9	2.9	32.4	
	Environmetal management Wate Act, SANS 10248	1	2.9	2.9	35.3	
	Gauteng Health Care Waste Management Regulation	2	5.9	5.9	41.2	
	Gauteng Health Care Waste Management Regulation, NEMWA	1	2.9	2.9	44.1	
	Gauteng Health Care Waste managemnt regulation	1	2.9	2.9	47.1	
	Gauteng health care waste regulation	3	8.8	8.8	55.9	
	Gauteng Health Care Waste Regulation	1	2.9	2.9	58.8	
	Geo-zone guiding manual, SOP, Health care waste regulation. Code of practice, circulars	1	2.9	2.9	61.8	
	health care waste policies and legislation	1	2.9	2.9	64.7	
	health care waste regulation	1	2.9	2.9	67.6	
	legislation, world health organization guidance and policies	1	2.9	2.9	70.6	
	National Enviornmental Mangement Act 107 of 1998,National Environmental Management: Waste Act 59 of 2008	1	2.9	2.9	73.5	
	National Health Act/ National Environmental Management Waste Act 2008/ Ntaional Environmental Management Act/ Road traffic Act/ Code of practice for health care waste	1	2.9	2.9	76.5	
	NEMA, OHS Act, National Helath Act	1	2.9	2.9	79.4	
	NEMWA, Gauteng Health care waste managemnent regulation	1	2.9	2.9	82.4	
	Occupational health and safety Act 85 of 1993 Gauteng Health Care Waste Mnagement Regulation Infection Prevention and Control principles	1	2.9	2.9	85.3	
	Occupational health and safety Act 85 of 1993 National Management Waste Act	1	2.9	2.9	88.2	
	OHS Act	1	2.9	2.9	91.2	
	OHS Act, National Health Act, NEMA, NEMWA	1	2.9	2.9	94.1	
	The NEMA Act of 2008, National waste strategy	1	2.9	2.9	97.1	
	waste management Act,Environmental Act,cradle to grave principle,Gauteng province waste management,SOP,Nationalcore standard	1	2.9	2.9	100.0	
	Total	34	100.0	100.0		


2.8. Has an integrated HCW committee been appointed at your HCF?	
	Frequency	Percent	Valid Percent	Cumulative Percent	
Valid	Yes	8	23.5	30.8	30.8	
	No	16	47.1	61.5	92.3	
	Uncertain	2	5.9	7.7	100.0	
	Total	26	76.5	100.0		
Missing	System	8	23.5			
Total	34	100.0			


Profession for member 1	
	Frequency	Percent	Valid Percent	Cumulative Percent	
Valid		26	76.5	76.5	76.5	
	Chief pharmacy	1	2.9	2.9	79.4	
	EHP	1	2.9	2.9	82.4	
	HCW Officer-Professional nurse	1	2.9	2.9	85.3	
	Health care waste officer	1	2.9	2.9	88.2	
	Infection prevention and Control coordinator	3	8.8	8.8	97.1	
	nursing manager	1	2.9	2.9	100.0	
	Total	34	100.0	100.0		


Profession for member 2	
	Frequency	Percent	Valid Percent	Cumulative Percent	
Valid		26	76.5	76.5	76.5	
	EHP	1	2.9	2.9	79.4	
	Infection prevention and control nurse	1	2.9	2.9	82.4	
	Logistics	1	2.9	2.9	85.3	
	Nursing service manager	1	2.9	2.9	88.2	
	OHS nurse	3	8.8	8.8	97.1	
	professional nurses	1	2.9	2.9	100.0	
	Total	34	100.0	100.0		


Profession for member 3	
	Frequency	Percent	Valid Percent	Cumulative Percent	
Valid		26	76.5	76.5	76.5	
	Clinical manager	1	2.9	2.9	79.4	
	EHP	1	2.9	2.9	82.4	
	Environmentalist	1	2.9	2.9	85.3	
	Health care risk waste officer	1	2.9	2.9	88.2	
	Infection prevention and contol nurse	1	2.9	2.9	91.2	
	OHS nurse	1	2.9	2.9	94.1	
	Quality Assurance coordinator	2	5.9	5.9	100.0	
	Total	34	100.0	100.0		


Profession for member 4	
	Frequency	Percent	Valid Percent	Cumulative Percent	
Valid		27	79.4	79.4	79.4	
	Cleaner	1	2.9	2.9	82.4	
	Cleaning department	1	2.9	2.9	85.3	
	Infection prevention and control nurse	1	2.9	2.9	88.2	
	Pharmacy manager	2	5.9	5.9	94.1	
	Procurement	1	2.9	2.9	97.1	
	Quality Assurance	1	2.9	2.9	100.0	
	Total	34	100.0	100.0		


Profession for member 5	
	Frequency	Percent	Valid Percent	Cumulative Percent	
Valid		27	79.4	79.4	79.4	
	Admin and logistics	1	2.9	2.9	82.4	
	Finance	1	2.9	2.9	85.3	
	Operational manager nursing	1	2.9	2.9	88.2	
	Quality assurance	1	2.9	2.9	91.2	
	Radiology head	2	5.9	5.9	97.1	
	Waste handlers	1	2.9	2.9	100.0	
	Total	34	100.0	100.0		


Profession for member 6	
	Frequency	Percent	Valid Percent	Cumulative Percent	
Valid		27	79.4	79.4	79.4	
	Cleaning services	1	2.9	2.9	82.4	
	Financial management	1	2.9	2.9	85.3	
	Nursing operational manager	1	2.9	2.9	88.2	
	OHS nurse	1	2.9	2.9	91.2	
	Quality assurance	1	2.9	2.9	94.1	
	skills and development	2	5.9	5.9	100.0	
	Total	34	100.0	100.0		


Profession for member 7	
	Frequency	Percent	Valid Percent	Cumulative Percent	
Valid		28	82.4	82.4	82.4	
	EHP	1	2.9	2.9	85.3	
	Infection Control	1	2.9	2.9	88.2	
	nursing manager	1	2.9	2.9	91.2	
	Porters	1	2.9	2.9	94.1	
	Procurement	2	5.9	5.9	100.0	
	Total	34	100.0	100.0		


Profession for member 8	
	Frequency	Percent	Valid Percent	Cumulative Percent	
Valid		29	85.3	85.3	85.3	
	Care takers	1	2.9	2.9	88.2	
	Environmental health officer	1	2.9	2.9	91.2	
	Environmental health officers	1	2.9	2.9	94.1	
	Facility manager	1	2.9	2.9	97.1	
	OHS nurse	1	2.9	2.9	100.0	
	Total	34	100.0	100.0		


2.10. Do the job descriptions of the Integrated HCW committee members include the management of HCW?ý Member 1	
	Frequency	Percent	Valid Percent	Cumulative Percent	
Valid	Yes	8	23.5	100.0	100.0	
Missing	System	26	76.5			
Total	34	100.0			


2.10. Do the job descriptions of the Integrated HCW committee members include the management of HCW?ý Member 2	
	Frequency	Percent	Valid Percent	Cumulative Percent	
Valid	Yes	7	20.6	87.5	87.5	
	No	1	2.9	12.5	100.0	
	Total	8	23.5	100.0		
Missing	System	26	76.5			
Total	34	100.0			


2.10. Do the job descriptions of the Integrated HCW committee members include the management of HCW?ý Member 3	
	Frequency	Percent	Valid Percent	Cumulative Percent	
Valid	Yes	5	14.7	71.4	71.4	
	No	1	2.9	14.3	85.7	
	Uncertain	1	2.9	14.3	100.0	
	Total	7	20.6	100.0		
Missing	System	27	79.4			
Total	34	100.0			


2.10. Do the job descriptions of the Integrated HCW committee members include the management of HCW?ý Member 4	
	Frequency	Percent	Valid Percent	Cumulative Percent	
Valid	Yes	6	17.6	85.7	85.7	
	No	1	2.9	14.3	100.0	
	Total	7	20.6	100.0		
Missing	System	27	79.4			
Total	34	100.0			


2.10. Do the job descriptions of the Integrated HCW committee members include the management of HCW?ý Member 5	
	Frequency	Percent	Valid Percent	Cumulative Percent	
Valid	Yes	5	14.7	71.4	71.4	
	No	1	2.9	14.3	85.7	
	Uncertain	1	2.9	14.3	100.0	
	Total	7	20.6	100.0		
Missing	System	27	79.4			
Total	34	100.0			


2.10. Do the job descriptions of the Integrated HCW committee members include the management of HCW?ý Member 6	
	Frequency	Percent	Valid Percent	Cumulative Percent	
Valid	Yes	4	11.8	66.7	66.7	
	No	2	5.9	33.3	100.0	
	Total	6	17.6	100.0		
Missing	System	28	82.4			
Total	34	100.0			


2.10. Do the job descriptions of the Integrated HCW committee members include the management of HCW?ý Member 7	
	Frequency	Percent	Valid Percent	Cumulative Percent	
Valid	Yes	3	8.8	42.9	42.9	
	No	2	5.9	28.6	71.4	
	Uncertain	2	5.9	28.6	100.0	
	Total	7	20.6	100.0		
Missing	System	27	79.4			
Total	34	100.0			


2.10. Do the job descriptions of the Integrated HCW committee members include the management of HCW?ý Member 8	
	Frequency	Percent	Valid Percent	Cumulative Percent	
Valid	Yes	4	11.8	80.0	80.0	
	Uncertain	1	2.9	20.0	100.0	
	Total	5	14.7	100.0		
Missing	System	29	85.3			
Total	34	100.0			


2.11. Have the Integrated HCW committee members received training? Member 1	
	Frequency	Percent	Valid Percent	Cumulative Percent	
Valid	Yes	8	23.5	100.0	100.0	
Missing	System	26	76.5			
Total	34	100.0			


2.11. Have the Integrated HCW committee members received training? Member 2	
	Frequency	Percent	Valid Percent	Cumulative Percent	
Valid	Yes	8	23.5	100.0	100.0	
Missing	System	26	76.5			
Total	34	100.0			


2.11. Have the Integrated HCW committee members received training? Member 3	
	Frequency	Percent	Valid Percent	Cumulative Percent	
Valid	Yes	8	23.5	100.0	100.0	
Missing	System	26	76.5			
Total	34	100.0			


2.11. Have the Integrated HCW committee members received training? Member 4	
	Frequency	Percent	Valid Percent	Cumulative Percent	
Valid	Yes	7	20.6	100.0	100.0	
Missing	System	27	79.4			
Total	34	100.0			


2.11. Have the Integrated HCW committee members received training? Member 5	
	Frequency	Percent	Valid Percent	Cumulative Percent	
Valid	Yes	7	20.6	100.0	100.0	
Missing	System	27	79.4			
Total	34	100.0			


2.11. Have the Integrated HCW committee members received training? Member 6	
	Frequency	Percent	Valid Percent	Cumulative Percent	
Valid	Yes	5	14.7	71.4	71.4	
	Uncertain	2	5.9	28.6	100.0	
	Total	7	20.6	100.0		
Missing	System	27	79.4			
Total	34	100.0			


2.11. Have the Integrated HCW committee members received training? Member 7	
	Frequency	Percent	Valid Percent	Cumulative Percent	
Valid	Yes	6	17.6	100.0	100.0	
Missing	System	28	82.4			
Total	34	100.0			


2.11. Have the Integrated HCW committee members received training? Member 8	
	Frequency	Percent	Valid Percent	Cumulative Percent	
Valid	Yes	5	14.7	100.0	100.0	
Missing	System	29	85.3			
Total	34	100.0			


2.12. If answered Yes to 2.11, please explain what type of training was received?	
	Frequency	Percent	Valid Percent	Cumulative Percent	
Valid		26	76.5	76.5	76.5	
	4 hour learning in health care waste managemnt by the service provider and the health  care waste officer	1	2.9	2.9	79.4	
	6 week distance health care waste management training, 4 hour health care waste management training, 1 hour seniour management of health care waste training	1	2.9	2.9	82.4	
	competence certificate in safet handling of health acre waste. Certificate for continuing professional development in health care waste training	1	2.9	2.9	85.3	
	formal training conducted by Geo-zone environmental and also informal waste trainings conducted by EHPs	1	2.9	2.9	88.2	
	formal, on-spot	1	2.9	2.9	91.2	
	Health care risk care waste training on segregation of waste that are generated in the facility/ facility	1	2.9	2.9	94.1	
	Health care waste managemnt by Geozone environmental	1	2.9	2.9	97.1	
	provide training on proper segregation of waste, personal protective equipment and ensure compliance with the use of the Act and regulations, report ofincidents in departments	1	2.9	2.9	100.0	
	Total	34	100.0	100.0		


2.13. How does the HCWMP gives direction to the internal collection, transport and storage of the following? Pathological/ Anatomical waste	
	Frequency	Percent	Valid Percent	Cumulative Percent	
Valid		7	20.6	20.6	20.6	
	All pathological waste should be disposed off in the red bucket for limbs kept in theatre or labour ward in locked freezer	1	2.9	2.9	23.5	
	Anatomical waste is collected from the wards to be stored in the murtary refridgerators and collected by the service provider	1	2.9	2.9	26.5	
	Anatomical waste is dicarded in red containers then placed in the refridgerator for collection	1	2.9	2.9	29.4	
	Anatomical waste is sored in the refridgerator within the ward during the shift (only in labour ward) then taken to the deport refridgerator. Other wards must bring in the anatomical waste immediately after generation	1	2.9	2.9	32.4	
	anatomical waste is stored in freezer and taken to stoarge area	1	2.9	2.9	35.3	
	Anatomical waste should be disposed in the red specicans and stored in the intermediate storage area in a freezer. From sluicetransported to the central storage bythe trolley	1	2.9	2.9	38.2	
	At maternity in freezer in sluice roo, general waste collector collects with trolley to maternity. Other units such as casualty unit to take to maternity in 24hrs and write in a book	1	2.9	2.9	41.2	
	Collector should wear a complete PPE to be transpoted on a leak proof trolley. To be stored in minus two degrees celcius/ freezer	1	2.9	2.9	44.1	
	Freezer, to be kept for less than 7 days	1	2.9	2.9	47.1	
	Includes tissues, organs, placentas and they are stored inside red anatomical specican from therefore are stored inside the freezer	1	2.9	2.9	50.0	
	It gives the standard on how and where to store before collection as well as what documents need to be filled in and followed up.	1	2.9	2.9	52.9	
	It is generated from maternity then taken tpo the to hospital central storage	1	2.9	2.9	55.9	
	Its stored in the lockable fridge, awaiting for service provider for collection	1	2.9	2.9	58.8	
	kept in a -2oc freezer amd service provider collects	1	2.9	2.9	61.8	
	kept in a freezer then taken to storage area	1	2.9	2.9	64.7	
	kept in freezer and service provider collects	1	2.9	2.9	67.6	
	Pathological anatomical waste is stored in the sluice room and on a daily basis it sent to the maternity for storage. The labour ward has a freezer which this waste is stored inside. Transportation is done using a trolley	1	2.9	2.9	70.6	
	pathological waste needs to be collected from the refridgeratosin the wards intermediate storage are and be stored in the 2degrees celcius freezer in the main storage area. Collection should be done using a wheelie trolley	1	2.9	2.9	73.5	
	Place in the red plastic in the red container and placed in the freezer in the central storage area	1	2.9	2.9	76.5	
	storage of pathological waste is at the freezer	1	2.9	2.9	79.4	
	stored in a leak proof container, labelled, collected and transported by trained waste handlers.	1	2.9	2.9	82.4	
	stored in a red bucket, collected with a steel trolley that is easy to clean. It is keptin a refridgerator until it is transported to themain storage area	1	2.9	2.9	85.3	
	stored in the refridgerator and service provider collects the pathological waste	1	2.9	2.9	88.2	
	Stored in the refridgerator and service provider collects the pathological waste	1	2.9	2.9	91.2	
	there is a standard operating procedure in place	1	2.9	2.9	94.1	
	This type pof waste collected from generation point e.g. maternity in the fridge (intermidiate storage). The medical waste trolley is used to transport anatomical waste from ward	1	2.9	2.9	97.1	
	waste is collected by service provider	1	2.9	2.9	100.0	
	Total	34	100.0	100.0		


2.13. How does the HCWMP gives direction to the internal collection, transport and storage of the following? Sharps (yellow container)	
	Frequency	Percent	Valid Percent	Cumulative Percent	
Valid		7	20.6	20.6	20.6	
	All sharps should be collected from the wards when they are 3/4 full, a trolley should be used to transport sharps from the wards to the central storage area.	1	2.9	2.9	23.5	
	All sharps waste is stored n the sluice room/ intermediate and then collected daily from the units to the waste storage area (Central storage area). A waste trolley is used to collect such waste	1	2.9	2.9	26.5	
	Filled/ closed sharps containers shopuld be moved to ward sluice room, then collecetd by waste collectors to mian storage	1	2.9	2.9	29.4	
	In sluice room general waste assistant removes to main storage area	1	2.9	2.9	32.4	
	Includes needles, sharps etc. they are  disposed inside yellow container, they are placed inside intermediate storage area. Thereafter waste collectors stores them at the medicalstoragearea	1	2.9	2.9	35.3	
	It directs you were and how long to use, where to store, how and when to close	1	2.9	2.9	38.2	
	It is generated from all wards, casualty and OPD and trolley is used and taken to storage area	1	2.9	2.9	41.2	
	It states that sharps container shpould not stay more than 90 days in the wards	1	2.9	2.9	44.1	
	kept in the sluice room, then taken to the storage are for service provider to collect	1	2.9	2.9	47.1	
	placed in sharps cointainer until 3/4 full. Taken to storage area, service provider collects	1	2.9	2.9	50.0	
	Sharp waste is collected from wards to the internal storage until collection by the service provider	1	2.9	2.9	52.9	
	Sharps container when 3/4 full must be put in sluice room and waste collectors collect it and store it in the deport	1	2.9	2.9	55.9	
	Sharps in yellow container	1	2.9	2.9	58.8	
	Sharps is stored in the wards until full then taken to the intermediate storage then central storage	1	2.9	2.9	61.8	
	Sharps must be labelled and closed when transported, stored and collected	1	2.9	2.9	64.7	
	Sharps waste collected from wards to the sluice room, then collected from the sluice through the central storage by the trolleys	1	2.9	2.9	67.6	
	storage of sharps is at the sluice room then taken to the storage area	1	2.9	2.9	70.6	
	stored in a yellow sharps container, properly labelled, colceted and transported by trained waste handlers to the main storage area prior to collection by the service provider. (trolley Is used to transport the sharps)	1	2.9	2.9	73.5	
	stored in the intermediate storage area for service provider to collect	1	2.9	2.9	76.5	
	Taken from wards to the sluice room and taken to the medical storage and weighed and collected by service provider	1	2.9	2.9	79.4	
	taken to storage area	1	2.9	2.9	82.4	
	there is a standard operating procedure in place	1	2.9	2.9	85.3	
	They are stored in the sluice room then taken to central storage and service provider collects	1	2.9	2.9	88.2	
	To be collected from a sluice room. Transported in a leak proof trolley to be stored in a lockable central storage	1	2.9	2.9	91.2	
	waste is collected by service provider	1	2.9	2.9	94.1	
	when it is full, it is placed in the sluice room whre it will be collected by waste colletors	1	2.9	2.9	97.1	
	Yellow sharps containers are used for disposal of sharps from wards waste is tored in the intermediate storage area and transported to central storage by trolley	1	2.9	2.9	100.0	
	Total	34	100.0	100.0		


2.13. How does the HCWMP gives direction to the internal collection, transport and storage of the following? Cytotoxic waste	
	Frequency	Percent	Valid Percent	Cumulative Percent	
Valid		25	73.5	73.5	73.5	
	N/A	1	2.9	2.9	76.5	
	stored in green bucket, collected and transported bytrained waste handlers to their storage area then service provider colects the cytotoxic waste.	1	2.9	2.9	79.4	
	stored in the intermediate storage area for the service provider to collect at the cytotoxic stprage area	1	2.9	2.9	82.4	
	Such waste isdispsoed inside a green cointaner whereby the unit will  label such cointainer with cytotoxic signage and it will therefore be stored at pharmacy	1	2.9	2.9	85.3	
	Taken from wards to the sluice room and taken to the medical storage and weighed and collected by service provider	1	2.9	2.9	88.2	
	taken to storage area	1	2.9	2.9	91.2	
	there is a standard operating procedure in place	1	2.9	2.9	94.1	
	To be collected in a lockable intermediate storage, to be transported to a central storage by leak proof trolle. To be collected on central storage by service provider	1	2.9	2.9	97.1	
	waste is collected by service provider	1	2.9	2.9	100.0	
	Total	34	100.0	100.0		


2.13. How does the HCWMP gives direction to the internal collection, transport and storage of the following? Pharmaceutical waste (green container)	
	Frequency	Percent	Valid Percent	Cumulative Percent	
Valid		8	23.5	23.5	23.5	
	All pharmaceutical waste is sent to pharmacy departemnt and service provider collect it from the pharmacy storage area	1	2.9	2.9	26.5	
	Are kept under a lockable storage and service provider collect themfrom pharmacy	1	2.9	2.9	29.4	
	In pharmacy, storage area, waste collects from pharmacy	1	2.9	2.9	32.4	
	It directs and guide you in when to remove the containers and where and how to store	1	2.9	2.9	35.3	
	It is generated at pharmacy	1	2.9	2.9	38.2	
	kept in the pharmacy for the service provider to collect	1	2.9	2.9	41.2	
	Must be taken to pharmacy with a form, professional nurse must take pharmaceutical to pharmacy. Service providercollect straight from pharmacy	1	2.9	2.9	44.1	
	Only kept in pharmacy, wards fill in a form for the collection of expired medication and pharmacy therefore collects ad thenit stored in pharmact till collection	1	2.9	2.9	47.1	
	Pharmaceutical disposed off in green containers then transported to facility pharmacy	1	2.9	2.9	50.0	
	Pharmaceutical waste in the green container in oor satelite pharmacy and to Dr George Mkari hospital storage area	1	2.9	2.9	52.9	
	Pharmaceutical waste is collected from pharmacy by the service provider after all the relevant collection requsts have been made	1	2.9	2.9	55.9	
	pharmaceutical waste is stored at pharmacy until service provider collects	1	2.9	2.9	58.8	
	Pharmaceutical waste is to be stored at the expired stock room at pharmacy and xall in the service provider to collect	1	2.9	2.9	61.8	
	pharmaceutical waste should be stored in the pharmacy department until removal is arranged with the service provider	1	2.9	2.9	64.7	
	placed in green cointainer at pharmacy. service provider collects	1	2.9	2.9	67.6	
	Stored at pharmacy in a lockable cage and awaiting for transportation and collection	1	2.9	2.9	70.6	
	stored at pharmacy until service provider collects the waste.	1	2.9	2.9	73.5	
	Stored in pharmacy ubder lock and jey. To be kept for 180 days	1	2.9	2.9	76.5	
	stored in the pharmacy then service provider collects	1	2.9	2.9	79.4	
	Stored inpharmacy until the service provider collects directly from the storage at pharmacy	1	2.9	2.9	82.4	
	storedin the pharmacy then service provider collects	1	2.9	2.9	85.3	
	Taken from wards to pharmacy for service provider to collect	1	2.9	2.9	88.2	
	taken to storage area	1	2.9	2.9	91.2	
	there is a standard operating procedure in place	1	2.9	2.9	94.1	
	waste is collected by service provider	1	2.9	2.9	97.1	
	Waste is stored in a separate storage area for pharmaceutical waste. The service provider collects directly from pharmacy	1	2.9	2.9	100.0	
	Total	34	100.0	100.0		


2.13. How does the HCWMP gives direction to the internal collection, transport and storage of the following? Infectious waste (red bag)	
	Frequency	Percent	Valid Percent	Cumulative Percent	
Valid		7	20.6	20.6	20.6	
	Collected daily in the sluice room to the main storage area	1	2.9	2.9	23.5	
	Collection from generation points e.g wards, casualty to the intermidiate storage area from intermediate to the central storage by the red waste bins	1	2.9	2.9	26.5	
	Collector to wear complete PPE, transport in a leak proof trolley. Stored in a central lockab,e storage	1	2.9	2.9	29.4	
	In the red plastic and placed in the cardboard bo wrapped and transported to the central storage area	1	2.9	2.9	32.4	
	In the wards it is removed twice a day and should not stay more than 7 days in the central storage area	1	2.9	2.9	35.3	
	Infectious waste is collected from wards by waste collectors to the internal storage area until collection by the service provider	1	2.9	2.9	38.2	
	infectious waste is stored in the wards until full then taken to the intermediate storage then central storage	1	2.9	2.9	41.2	
	Infectious waste will be removed from the wards by the internal waste collectors using the trolley. All infectious waste will be stored in the central storage area until collection is done by the service provider	1	2.9	2.9	44.1	
	It directs you on how to mark, close and remove containers	1	2.9	2.9	47.1	
	It is generated at all wards, casualty and OPD and taken to storage area	1	2.9	2.9	50.0	
	kept in red liners, service provider collects	1	2.9	2.9	52.9	
	kept in the sluice room, then taken to the storage are for service provider to collect	1	2.9	2.9	55.9	
	Once filled, tie the bag and place it in to wheelei bin, once tge wheelie bin is full send it to sluice room for collcetion to main storage	1	2.9	2.9	58.8	
	Red liners for disposal of medical waste from sluice room waste is transported to the central storage. Service provider collects waste from central storage area	1	2.9	2.9	61.8	
	Sluice room, wheelie bin waste general assistant take to main storage area	1	2.9	2.9	64.7	
	storage of infectious waste is at the sluice room then taen to the storag area	1	2.9	2.9	67.6	
	Stored in intermediate storage when full then it is collected by waste transporters	1	2.9	2.9	70.6	
	stored in red liner at the sluice room in the wards. Collected and transported by trained waste handlers to the main storage area for service provider to collect.	1	2.9	2.9	73.5	
	stored in the intermediate storage area for service provider to collect	1	2.9	2.9	76.5	
	Taken from wards to the sluice room and taken to the medical storage and weighed and collected by service provider	1	2.9	2.9	79.4	
	taken to storage area	1	2.9	2.9	82.4	
	there is a standard operating procedure in place	1	2.9	2.9	85.3	
	They are stored in the sluice room then taken to central storage and service provider collects	1	2.9	2.9	88.2	
	This include soiled nappies where by red plastic is used when its full and stored at intermediate storage. Waste colectors will therefore fetch it from intermediate to medical storage	1	2.9	2.9	91.2	
	waste is collected by service provider	1	2.9	2.9	94.1	
	Waste is stored in the sluice room and daily it is collected and transported in a waste trolley to be stored at the central storage area	1	2.9	2.9	97.1	
	When full the cleaner seals it with a hazardous tape and put it at sluice room then the health care waste collector collects it and put it in deport	1	2.9	2.9	100.0	
	Total	34	100.0	100.0		


2.13. How does the HCWMP gives direction to the internal collection, transport and storage of the following? Radioactive waste	
	Frequency	Percent	Valid Percent	Cumulative Percent	
Valid		21	61.8	61.8	61.8	
	First to be stored in a led bion to decay radioactivity. Collected to central storage in a leak prrof trolle. Stored in a lockable central storage	1	2.9	2.9	64.7	
	N/A	1	2.9	2.9	67.6	
	Not generated	1	2.9	2.9	70.6	
	Only handled in X-ray department and collected on site	1	2.9	2.9	73.5	
	Radioactive waste is collected and disposed of in plastic bags and sold to a service provider	1	2.9	2.9	76.5	
	Radioactive waste is collected and stored in the storage area in the unit. The service provider collects directly from the units	1	2.9	2.9	79.4	
	stored at radographey departement area for service provider to collect	1	2.9	2.9	82.4	
	Such waste include CT scanner, nuclear medicine service provider whereby radiology department has a service provider that collect such waste	1	2.9	2.9	85.3	
	taken to storage area	1	2.9	2.9	88.2	
	there is a standard operating procedure in place	1	2.9	2.9	91.2	
	They are stored in the shelves and they recycle them	1	2.9	2.9	94.1	
	waste is collected by service provider	1	2.9	2.9	97.1	
	We use digital x-rays	1	2.9	2.9	100.0	
	Total	34	100.0	100.0		


2.13. How does the HCWMP gives direction to the internal collection, transport and storage of the following? General waste	
	Frequency	Percent	Valid Percent	Cumulative Percent	
Valid		7	20.6	20.6	20.6	
	All the household waste is being collected from all the wards with black general waste bins straight to the storage area and to be collcetd by municiplaity	1	2.9	2.9	23.5	
	Black/ transperant liner are used to dispose general waste. The waste is collected by municipality	1	2.9	2.9	26.5	
	black/transperant liner is used as a means of container. Collected and transported to the main storage area for the unicipality to collect.	1	2.9	2.9	29.4	
	Black/transperant liners are used. Waste transported to skip area with a trolley	1	2.9	2.9	32.4	
	Cleaners put the geenral waste in sluice room, then general waste collector collects and gp throw it in a skip bin	1	2.9	2.9	35.3	
	Collected by general assistants from different departments, transported using a bakkie to the main storage area/ skip	1	2.9	2.9	38.2	
	General waste is stored in the wards until full then taken to the intermediate storage then central storage	1	2.9	2.9	41.2	
	general waste is transported to the central storage disposal siteusing wheelie bins and trolleys. General waste is  kept in the skip bins	1	2.9	2.9	44.1	
	In the vblack plastic or transparent plastic and transported by a bakkie to central storage	1	2.9	2.9	47.1	
	Includes left over foods and drinks, paper, flowers.  Generated through administration and it is collected by trolley from all area and collected by municipality	1	2.9	2.9	50.0	
	is collected from different area and taken to the storage area foe collection by the municipality	1	2.9	2.9	52.9	
	Is stored at general waste area and is transported by municipal waste truck	1	2.9	2.9	55.9	
	It guide you in where to store general waste, how to keep rodents out etc	1	2.9	2.9	58.8	
	It is collceted once a week from the hospital premises by the service provider but in the wards it is collected twice a day	1	2.9	2.9	61.8	
	kept in the sluice room then taken to the skip	1	2.9	2.9	64.7	
	kept in the sluice room, then taken to the storage are for municipality to collect	1	2.9	2.9	67.6	
	Kept in transparent liners. Municipal collects	1	2.9	2.9	70.6	
	Sluice room to be collected by general waste assistants to skip	1	2.9	2.9	73.5	
	stored in the intermediate storage area for service provider to collect	1	2.9	2.9	76.5	
	Stored in the sluice room then when it is full waste transporters dispose of it in the skip bins	1	2.9	2.9	79.4	
	Taken from wards to the sluice room and taken to the medical storage and weighed and collected by municipality	1	2.9	2.9	82.4	
	taken to storage area	1	2.9	2.9	85.3	
	there is a standard operating procedure in place	1	2.9	2.9	88.2	
	They are stored in the sluice room then taken to central storage and service provider collects	1	2.9	2.9	91.2	
	To be collectted in 30 minutes intervals. Transported in a marcked trolley. Stored in compacted bins at central storage	1	2.9	2.9	94.1	
	waste is collected by municipality	1	2.9	2.9	97.1	
	Waste is stored in the sluice room and collected on a daily basis from the sluice room. It is sent to the central storage	1	2.9	2.9	100.0	
	Total	34	100.0	100.0		


2.13. How does the HCWMP gives direction to the internal collection, transport and storage of the following? Chemical waste	
	Frequency	Percent	Valid Percent	Cumulative Percent	
Valid		14	41.2	41.2	41.2	
	Collector should wear a complete PPE use leak prrof trolley to transport store in a lockabe central storage	1	2.9	2.9	44.1	
	Extra ordinary waste such as flourescent tubes are collected from the units during replacements and stored in a box and arrangements are made with the service provider for collection	1	2.9	2.9	47.1	
	Include all discarded solid, liquid and gaseous chemica;	1	2.9	2.9	50.0	
	Is replaced in the same container by which it was initially supplied, for return to the supplier of the product	1	2.9	2.9	52.9	
	kept in the sluice room, then taken to the storage are for service provider to collect	1	2.9	2.9	55.9	
	N/A	1	2.9	2.9	58.8	
	Not sure	1	2.9	2.9	61.8	
	Returned back to manufacturer/ supplier for disposal	1	2.9	2.9	64.7	
	Returned to supplier	1	2.9	2.9	67.6	
	Service provider is called when the need arises e.g. flourescent tubes and batteries	1	2.9	2.9	70.6	
	stored at the wards until. Collection and transportation will be determined by the contents.	1	2.9	2.9	73.5	
	stored in the intermediate storage area for service provider to collect	1	2.9	2.9	76.5	
	Taken back to the supplier	1	2.9	2.9	79.4	
	taken to storage area	1	2.9	2.9	82.4	
	there is a standard operating procedure in place	1	2.9	2.9	85.3	
	They are stored in the sluice room then taken to central storage and service provider collects	1	2.9	2.9	88.2	
	Transported to facility`s central storage and specicans are collected by service provider	1	2.9	2.9	91.2	
	User green container (pharmaceutical container) to contain the waste, then label the container as chemical waste and attach the MSDS on the container	1	2.9	2.9	94.1	
	waste is collected by service provider	1	2.9	2.9	97.1	
	X-ray has store room. Private company phoned to collect	1	2.9	2.9	100.0	
	Total	34	100.0	100.0		


2.14. Is there a designated person/s responsible for HCW collection and storage within the HCF?	
	Frequency	Percent	Valid Percent	Cumulative Percent	
Valid	Yes	27	79.4	100.0	100.0	
Missing	System	7	20.6			
Total	34	100.0			


2.15. Have the designated person/s responsible for collection and storage of HCW received any training?	
	Frequency	Percent	Valid Percent	Cumulative Percent	
Valid	Yes	27	79.4	100.0	100.0	
Missing	System	7	20.6			
Total	34	100.0			


2.16. If answered Yes to 2.15, please explain the type of training received.	
	Frequency	Percent	Valid Percent	Cumulative Percent	
Valid		7	20.6	20.6	20.6	
	4 hour training	1	2.9	2.9	23.5	
	4 hour traning by service provider and daily on spot training	1	2.9	2.9	26.5	
	All from handling waste, transporting and dangers of not wearing PPE	1	2.9	2.9	29.4	
	Continuous healthcare risk waste training	1	2.9	2.9	32.4	
	formal training by service provider and induction training was done by health care waste officer	1	2.9	2.9	35.3	
	formal training facilitated by Geo-zone environmental and informal training done by environmental health practitioners	1	2.9	2.9	38.2	
	formal, on-spot	1	2.9	2.9	41.2	
	Geo-zone training	1	2.9	2.9	44.1	
	Geo-zone training and internal training from EHP	1	2.9	2.9	47.1	
	handling, storage and collection of waste in a comprehensive mnner which includes the oocupational hazards that are involved	1	2.9	2.9	50.0	
	hazardous waste managemnt	1	2.9	2.9	52.9	
	Health care risk waste includes procedures, guidelines on how to collect waste type of transportation used	1	2.9	2.9	55.9	
	health care waste managemnt training (storage, collection and transportation of health care waste . The health hazards related to the handling of health care waste.	1	2.9	2.9	58.8	
	health care waste training by Geo-zone and central office	1	2.9	2.9	61.8	
	health care wastevtraining for 4 hours	1	2.9	2.9	64.7	
	Hnadling, collection and transportation of waste (external). Segregation (internal) and PPE for waste collcetors (internal)	1	2.9	2.9	67.6	
	How waste must be segregated ad which consumables must be used	1	2.9	2.9	70.6	
	In service training was given by health care watse officer also attended Geo-zone training at the facility	1	2.9	2.9	73.5	
	Internal training by infection control nurse and EHP. Through Health Care waste awareness in the facility. By external trainers	1	2.9	2.9	76.5	
	Level 1 to level 4 training in safe handling of health care waste that lasts up to 4 hours	1	2.9	2.9	79.4	
	management of health care waste training, detailed training on how waste is generated until its disposal, managemnt of spillages of waste within the health care facility, detailed training on procedures to follow when handling spillages, training on the use of personal protective equipment and occupational health and safety (in regards to injuries)	1	2.9	2.9	82.4	
	NQF1	1	2.9	2.9	85.3	
	Received four hours training by the service provider	1	2.9	2.9	88.2	
	Theoritical and practical health care waste training	1	2.9	2.9	91.2	
	Training by Geo-zone environmental	1	2.9	2.9	94.1	
	Training on how to wear PPE, how to handle waste and how to load the waste to the trolleys and how to transport	1	2.9	2.9	97.1	
	waste management	1	2.9	2.9	100.0	
	Total	34	100.0	100.0		


2.17. What is the designated person been appointed as?	
	Frequency	Percent	Valid Percent	Cumulative Percent	
Valid		7	20.6	20.6	20.6	
	1	1	2.9	2.9	23.5	
	cleaner	6	17.6	17.6	41.2	
	Cleaner	4	11.8	11.8	52.9	
	general assistant	8	23.5	23.5	76.5	
	General assistant	4	11.8	11.8	88.2	
	General worker	1	2.9	2.9	91.2	
	Health care waste collector	1	2.9	2.9	94.1	
	waste collector	1	2.9	2.9	97.1	
	Waste collector	1	2.9	2.9	100.0	
	Total	34	100.0	100.0		


2.18. What type of employment is it?	
	Frequency	Percent	Valid Percent	Cumulative Percent	
Valid	Full time	26	76.5	100.0	100.0	
Missing	System	8	23.5			
Total	34	100.0			


2.18. What type of employment is it? Other (specify) _________________	
	Frequency	Percent	Valid Percent	Cumulative Percent	
Valid	1	4	11.8	100.0	100.0	
Missing	System	30	88.2			
Total	34	100.0			


2.19. Does your HCWMP deal with the following? Reduction of  generation rate of HCW	
	Frequency	Percent	Valid Percent	Cumulative Percent	
Valid	Yes	25	73.5	92.6	92.6	
	No	2	5.9	7.4	100.0	
	Total	27	79.4	100.0		
Missing	System	7	20.6			
Total	34	100.0			


2.19. Does your HCWMP deal with the following? Promote better segregation	
	Frequency	Percent	Valid Percent	Cumulative Percent	
Valid	Yes	25	73.5	100.0	100.0	
Missing	System	9	26.5			
Total	34	100.0			


2.19. Does your HCWMP deal with the following? Encourage staff training	
	Frequency	Percent	Valid Percent	Cumulative Percent	
Valid	Yes	27	79.4	100.0	100.0	
Missing	System	7	20.6			
Total	34	100.0			


2.19. Does your HCWMP deal with the following? Facilitate proper storage	
	Frequency	Percent	Valid Percent	Cumulative Percent	
Valid	Yes	27	79.4	100.0	100.0	
Missing	System	7	20.6			
Total	34	100.0			


2.19. Does your HCWMP deal with the following? Ensure procurement of goods	
	Frequency	Percent	Valid Percent	Cumulative Percent	
Valid	Yes	26	76.5	96.3	96.3	
	Uncertain	1	2.9	3.7	100.0	
	Total	27	79.4	100.0		
Missing	System	7	20.6			
Total	34	100.0			


2.19. Does your HCWMP deal with the following? Provision of effective transportation	
	Frequency	Percent	Valid Percent	Cumulative Percent	
Valid	Yes	26	76.5	96.3	96.3	
	No	1	2.9	3.7	100.0	
	Total	27	79.4	100.0		
Missing	System	7	20.6			
Total	34	100.0			


2.19. Does your HCWMP deal with the following? Ensure effective Treatment and disposal	
	Frequency	Percent	Valid Percent	Cumulative Percent	
Valid	Yes	25	73.5	96.2	96.2	
	No	1	2.9	3.8	100.0	
	Total	26	76.5	100.0		
Missing	System	8	23.5			
Total	34	100.0			


2.20. Is there a system that ensures retrievability and accessibility (documentation control) of HCW documentation?	
	Frequency	Percent	Valid Percent	Cumulative Percent	
Valid	Yes	21	61.8	77.8	77.8	
	No	4	11.8	14.8	92.6	
	Uncertain	2	5.9	7.4	100.0	
	Total	27	79.4	100.0		
Missing	System	7	20.6			
Total	34	100.0			


2.21. Does the HCWMP make provision for emergency conditions? (e.g. shortage of consumables.)	
	Frequency	Percent	Valid Percent	Cumulative Percent	
Valid	Yes	24	70.6	88.9	88.9	
	No	2	5.9	7.4	96.3	
	Uncertain	1	2.9	3.7	100.0	
	Total	27	79.4	100.0		
Missing	System	7	20.6			
Total	34	100.0			


2.22. How does your HCF address HCW shortfalls that may need budget?	
	Frequency	Percent	Valid Percent	Cumulative Percent	
Valid		8	23.5	23.5	23.5	
	At the beginning of the new financial year the waste department is requested to submit a cost center plan as part of a contigency plan in cases of shortfalls that may need budget	1	2.9	2.9	26.5	
	Budget allocated for health care waste consumables	1	2.9	2.9	29.4	
	Budget is requested	1	2.9	2.9	32.4	
	By requesting additional funds from the hospital budget	1	2.9	2.9	35.3	
	communication is sent to the head office in request for assistance, should the health care facility be able to acquire cost/finance to resolve the shortfall	1	2.9	2.9	38.2	
	consultaion with CEO	1	2.9	2.9	41.2	
	Does not make provision	1	2.9	2.9	44.1	
	Fill out the form and take it to procurement department	1	2.9	2.9	47.1	
	health care facility does not handle as payments made by province	1	2.9	2.9	50.0	
	management is requested to approve the budget	1	2.9	2.9	52.9	
	Motivation letter is subitted for approval	1	2.9	2.9	55.9	
	Motivation letter with proof of quatation. We use petty cash to buy	1	2.9	2.9	58.8	
	motivations are sent to managemnt for approval	1	2.9	2.9	61.8	
	Negotiate with finance department, write a motivation for your needs and wait wait for approval. If it is something that requires less money, e.h less than R2000.00 petty cash will be used	1	2.9	2.9	64.7	
	Never experinced shortfalls	1	2.9	2.9	67.6	
	not certain	1	2.9	2.9	70.6	
	Not expirienced	1	2.9	2.9	73.5	
	Not sure	1	2.9	2.9	76.5	
	So far we wait until approval is granted by finance	1	2.9	2.9	79.4	
	The HCF has a separate budget allocated for such short falls. To access motivation must be written	1	2.9	2.9	82.4	
	The health care facility ensures that there is enough budget that is allocated foe health care waste annually, so shortfalls has never happened due to budget contraints	1	2.9	2.9	85.3	
	The health care facility has not experienced any shortfalls that required a budget	1	2.9	2.9	88.2	
	Through managemnt meetings and reporting to the relevant departments that workhand in hand with health care waste.	1	2.9	2.9	91.2	
	through motivation	1	2.9	2.9	94.1	
	uncertain	1	2.9	2.9	97.1	
	yes	1	2.9	2.9	100.0	
	Total	34	100.0	100.0		


3.1. Does the HCWMP include health and safety hazards and risks from HCW point of generation until it leaves your facility?	
	Frequency	Percent	Valid Percent	Cumulative Percent	
Valid	Yes	25	73.5	92.6	92.6	
	No	2	5.9	7.4	100.0	
	Total	27	79.4	100.0		
Missing	System	7	20.6			
Total	34	100.0			


3.2. Is there a procedure in place for reporting of injuries related to HCW?	
	Frequency	Percent	Valid Percent	Cumulative Percent	
Valid	Yes	27	79.4	100.0	100.0	
Missing	System	7	20.6			
Total	34	100.0			


3.3. Do you review the effectiveness of the health and safety measures?	
	Frequency	Percent	Valid Percent	Cumulative Percent	
Valid	Yes	19	55.9	73.1	73.1	
	No	1	2.9	3.8	76.9	
	Uncertain	6	17.6	23.1	100.0	
	Total	26	76.5	100.0		
Missing	System	8	23.5			
Total	34	100.0			


3.4. If answered Yes at question 3.3 indicate the revision frequency of the health and safety measures?	
	Frequency	Percent	Valid Percent	Cumulative Percent	
Valid	After every incident	12	35.3	60.0	60.0	
	Bi-annually (every 6 months)	2	5.9	10.0	70.0	
	Annually (once a year)	3	8.8	15.0	85.0	
	Every 2 years	2	5.9	10.0	95.0	
	6	1	2.9	5.0	100.0	
	Total	20	58.8	100.0		
Missing	System	14	41.2			
Total	34	100.0			


3.5. Indicate if the following aspects are complied with regarding Health Care Risk Waste (HCRW) management at your HCF? Health and safety policy	
	Frequency	Percent	Valid Percent	Cumulative Percent	
Valid	Yes	21	61.8	77.8	77.8	
	No	2	5.9	7.4	85.2	
	Uncertain	4	11.8	14.8	100.0	
	Total	27	79.4	100.0		
Missing	System	7	20.6			
Total	34	100.0			


3.5. Indicate if the following aspects are complied with regarding Health Care Risk Waste (HCRW) management at your HCF? Appointment of Health and safety representatives	
	Frequency	Percent	Valid Percent	Cumulative Percent	
Valid	Yes	25	73.5	92.6	92.6	
	No	1	2.9	3.7	96.3	
	Uncertain	1	2.9	3.7	100.0	
	Total	27	79.4	100.0		
Missing	System	7	20.6			
Total	34	100.0			


3.5. Indicate if the following aspects are complied with regarding Health Care Risk Waste (HCRW) management at your HCF? Training of health and safety representatives	
	Frequency	Percent	Valid Percent	Cumulative Percent	
Valid	Yes	22	64.7	81.5	81.5	
	No	2	5.9	7.4	88.9	
	Uncertain	3	8.8	11.1	100.0	
	Total	27	79.4	100.0		
Missing	System	7	20.6			
Total	34	100.0			


3.5. Indicate if the following aspects are complied with regarding Health Care Risk Waste (HCRW) management at your HCF? Induction training of workers	
	Frequency	Percent	Valid Percent	Cumulative Percent	
Valid	Yes	25	73.5	92.6	92.6	
	No	1	2.9	3.7	96.3	
	Uncertain	1	2.9	3.7	100.0	
	Total	27	79.4	100.0		
Missing	System	7	20.6			
Total	34	100.0			


3.5. Indicate if the following aspects are complied with regarding Health Care Risk Waste (HCRW) management at your HCF? Disaster/ Emergency plan	
	Frequency	Percent	Valid Percent	Cumulative Percent	
Valid	Yes	23	67.6	85.2	85.2	
	No	1	2.9	3.7	88.9	
	Uncertain	3	8.8	11.1	100.0	
	Total	27	79.4	100.0		
Missing	System	7	20.6			
Total	34	100.0			


3.5. Indicate if the following aspects are complied with regarding Health Care Risk Waste (HCRW) management at your HCF? Health risk assessments conducted	
	Frequency	Percent	Valid Percent	Cumulative Percent	
Valid	Yes	23	67.6	88.5	88.5	
	No	2	5.9	7.7	96.2	
	Uncertain	1	2.9	3.8	100.0	
	Total	26	76.5	100.0		
Missing	System	8	23.5			
Total	34	100.0			


3.6. Please indicate the extent to which the following statements listed below are considered a priority at your HCF? Conducting inspections	
	Frequency	Percent	Valid Percent	Cumulative Percent	
Valid	Higher priority	27	79.4	100.0	100.0	
Missing	System	7	20.6			
Total	34	100.0			


3.6. Please indicate the extent to which the following statements listed below are considered a priority at your HCF? Feedback regarding inspection findings	
	Frequency	Percent	Valid Percent	Cumulative Percent	
Valid	2	27	79.4	100.0	100.0	
Missing	System	7	20.6			
Total	34	100.0			


3.6. Please indicate the extent to which the following statements listed below are considered a priority at your HCF? Education and providing information	
	Frequency	Percent	Valid Percent	Cumulative Percent	
Valid	1	2	5.9	7.4	7.4	
	2	25	73.5	92.6	100.0	
	Total	27	79.4	100.0		
Missing	System	7	20.6			
Total	34	100.0			


4.1. What is your understanding of the following environmental principles which are applicable to HCW management at your HCF? The polluter pays principle	
	Frequency	Percent	Valid Percent	Cumulative Percent	
Valid		12	35.3	35.3	35.3	
	Every generator of waste is entitled to pay for waste generated	1	2.9	2.9	38.2	
	Fines to non complaint users when ermitting harmful emmissions that exceed limits	1	2.9	2.9	41.2	
	For every waste you generate, you must pay for it	1	2.9	2.9	44.1	
	If you pollute the environment, you will pay by means of climate change.	1	2.9	2.9	47.1	
	If you pollute you will pay	1	2.9	2.9	50.0	
	It is an economic idea that firms or consumers should pay for the cost of thr negative impact or pollution they cause within the environment.	1	2.9	2.9	52.9	
	It means if you are a polluter, it is your responsibility to ensure that cost of disposal is covered and that the waste does not harm the environment or humankind	1	2.9	2.9	55.9	
	It means that any organisation that causes pollution that organasation has to pay cost or cleaning it u or that if the institution does not segregate waste prorpely and causes pollution it is that institution`s responsibility to pay	1	2.9	2.9	58.8	
	Means that any organisation that is causing pollution is liabe for the costs pf the cleaning it up. Therefore if pollution results from poor management of health care waste then the organisation running the health care facility	1	2.9	2.9	61.8	
	Should any waste from the hospital be found dumped anywhere in South Africa, the hospital will be responsible for all costs included in its removal and disposal	1	2.9	2.9	64.7	
	States that who ever pollutes is personally liable according to NEMA	1	2.9	2.9	67.6	
	The facility pays forremoval/destruction of waste	1	2.9	2.9	70.6	
	The generator is liable for any incidences that may occur.	1	2.9	2.9	73.5	
	The generator is responsible for any pollution that may occur	1	2.9	2.9	76.5	
	The party responsible for producing pollution is responsible for paying for the damage done to the environment	1	2.9	2.9	79.4	
	The polluter or any one emmiting waste/ pollutants in the environment needs to apply for emission lisvence and also pay for the emissions	1	2.9	2.9	82.4	
	The polluter pays principle means that any organisation/ facility causing pollution is liable for the costs of cleaning it up	1	2.9	2.9	85.3	
	The polluter will pay if they pollute for example the sea	1	2.9	2.9	88.2	
	The poluter pays principle is enacted to make the party responisble for producing helath care waste, responsible for paying. That means as the waste generators, our facility is responsible to pay for treating our waste	1	2.9	2.9	91.2	
	The principle states that everyone who is responsible for generating waste should ensure that the waste is disposed and managed properly and if so the person will haveto pay a fine	1	2.9	2.9	94.1	
	This means that the generator of the waste is responible for handling and disposing waste produced by him/ her and liable for any damage caused to the environment or human health from the waste generated.	1	2.9	2.9	97.1	
	Those who pollute must be resposible for cost of damage	1	2.9	2.9	100.0	
	Total	34	100.0	100.0		


4.1. What is your understanding of the following environmental principles which are applicable to HCW management at your HCF? The principle of green procurement	
	Frequency	Percent	Valid Percent	Cumulative Percent	
Valid		14	41.2	41.2	41.2	
	Buying produts that are recycable, last longer or produce less waste	1	2.9	2.9	44.1	
	Buying/ procuring of materials that are recyclable or reusable. Materials that have less harm to the environment.	1	2.9	2.9	47.1	
	Eac institution should procure products that are friendly to the environment to reduce carbon foot prints	1	2.9	2.9	50.0	
	Green procurement is when an organisation makes a commitment of purchasing goods that are protective of the environment	1	2.9	2.9	52.9	
	Health care facility should reduce and recycle	1	2.9	2.9	55.9	
	Institutions should procure environmentally friendly products.	1	2.9	2.9	58.8	
	It is the principle that guides procurement in to purchasing resources or services that has a less negative impact on the environment	1	2.9	2.9	61.8	
	It means that an institution should buy products that are environmentally friendly.	1	2.9	2.9	64.7	
	Its when organisation makes a commitment to purchase goods thatare protective of the environment	1	2.9	2.9	67.6	
	Means that the institution is procurement must be commited in purchasing goods that are protective of the environment	1	2.9	2.9	70.6	
	Paying for products and services that have least impact on the environment	1	2.9	2.9	73.5	
	Procure or purchase products in reusable or recycable containers to reduce waste	1	2.9	2.9	76.5	
	Procurement of environmrnta friendly material	1	2.9	2.9	79.4	
	purchasing recycled products thatare bio-degradable	1	2.9	2.9	82.4	
	Purchasing recycled products thatare bio-degradable	1	2.9	2.9	85.3	
	The principle emphaiszes on purchasing of green products or products that don2t have a negative impact on the ebvironment or contribute to global warming	1	2.9	2.9	88.2	
	The procurement of environmentakky friendly health care waste consumables that will not cause harm to the environment	1	2.9	2.9	91.2	
	The purchase of environmentally friendly producers and services. This procedure is against pollution or activities that are related to pollution or negative impact on the environment.	1	2.9	2.9	94.1	
	This means considering the costs of securing health care waste materials and manufacturing, transporting, storing, handling and disposal of health care waste products	1	2.9	2.9	97.1	
	This principle states the purchase of producs tha are less hazardous to the environment with no negative impacts to the environment	1	2.9	2.9	100.0	
	Total	34	100.0	100.0		


4.1. What is your understanding of the following environmental principles which are applicable to HCW management at your HCF? The precautionary principle	
	Frequency	Percent	Valid Percent	Cumulative Percent	
Valid		19	55.9	55.9	55.9	
	A strategy to cope with possible risks, e.g. risks of nano technology, genetically modified organisms and systemic insecticides.	1	2.9	2.9	58.8	
	All waste shouldbe handled in such a manner that it is assumed that it can acuse infection	1	2.9	2.9	61.8	
	Always assume that waste is hazardous until shown to be safe. This means that where it is unknown whether a waste contain hazardous substances it is important to take all the necessary precaution and treat it as if is hazardous.	1	2.9	2.9	64.7	
	Ensuring a healthy and safe environment by setting up rules/legislations that governs people on how to conduct or handle waste	1	2.9	2.9	67.6	
	Institutions should always be incognisense of their pollution	1	2.9	2.9	70.6	
	Is a strategy to cope with possible risks where scientific understanding is incomplete	1	2.9	2.9	73.5	
	It is a n approach to risk management that has been developed in circumstances of scientific uncertainty	1	2.9	2.9	76.5	
	It is when there is a threat to the environment precautionary measures should be taken even if they are not scientific precautionery measures	1	2.9	2.9	79.4	
	precautionary principle is a strategy to cope with possible risks where scientific understanding is yet incomplete, policies and Acts shpould be used in the health care facility toavoid risks	1	2.9	2.9	82.4	
	Take care of equipment etc	1	2.9	2.9	85.3	
	The generator of waste should ensure that waste is treatedat a close proximity and clean as possible.	1	2.9	2.9	88.2	
	The precautionary principle is when the generator always assumes that waste is hazardous until shown to be safe	1	2.9	2.9	91.2	
	The principle emphasizes on safety precautions that should be followed to avoid injuries that may related to poor waste management or that may relate to occupational hazards	1	2.9	2.9	94.1	
	This means before decisions are made on waste managemnt consideration is dne on the costs and impacts of such decision	1	2.9	2.9	97.1	
	This means that waste is hazardous until shown to be safe. Means that when handling waste is necessary to take precautionay measures and treat it as if is hazardous	1	2.9	2.9	100.0	
	Total	34	100.0	100.0		


4.1. What is your understanding of the following environmental principles which are applicable to HCW management at your HCF? Duty of care	
	Frequency	Percent	Valid Percent	Cumulative Percent	
Valid		18	52.9	52.9	52.9	
	A requiremen that a health care waste officer act towards others e.g. staff and payients to ensure that they are free from harm	1	2.9	2.9	55.9	
	All waste should be handled in such a manner that it will not create harm or injuries to other people	1	2.9	2.9	58.8	
	Any organisation that generates waste has a duty to dispose of the wast safely. This means health care facilities must take responsibility for the disposal of the waste that it generated.	1	2.9	2.9	61.8	
	Caring for the environment, the community and those responsible for waste management. Ensuring a healthy and safe environment	1	2.9	2.9	64.7	
	Duty of care means that only organisation that generates waste has a duty to disposed of the waste safely	1	2.9	2.9	67.6	
	Health care facility is responsible for handling and managing waste	1	2.9	2.9	70.6	
	Is a legal obligation to ensure the safety of others	1	2.9	2.9	73.5	
	It is a standard in the flow of negligence where a person is to act like a responsible person in a given circumstances	1	2.9	2.9	76.5	
	It is everyone duty to take care	1	2.9	2.9	79.4	
	Its imposed on an individual requiring that person to adhere to standards and regulations= with perfoming any acts.	1	2.9	2.9	82.4	
	Legal obligation to ensure that safety to others	1	2.9	2.9	85.3	
	not certain	1	2.9	2.9	88.2	
	Responsibility to avoid acts that may cause harm to others	1	2.9	2.9	91.2	
	The duty of carerelates to protecting the environment and ensuring that waste that is generated is properly disposed	1	2.9	2.9	94.1	
	This means that all the insitution must take responsibilities for the  disposal of the waste they generate	1	2.9	2.9	97.1	
	This means the generator of waste is responsible for the waste generated and should ensure its not hazardous to human health or the environment	1	2.9	2.9	100.0	
	Total	34	100.0	100.0		


4.1. What is your understanding of the following environmental principles which are applicable to HCW management at your HCF? Cradle to grave principle	
	Frequency	Percent	Valid Percent	Cumulative Percent	
Valid		11	32.4	32.4	32.4	
	Accountability of the generator for their wastefrom the point of generation to the point of disposal	1	2.9	2.9	35.3	
	Clean up the environment, the community and those responisble for waste managemnt. Esuring a healthy and safe environment.	1	2.9	2.9	38.2	
	Cradle to grave principle explains the safe managemnt of health care waste management from point of generation to disposal	1	2.9	2.9	41.2	
	cradle to grave principle refers to waste managemnt from point of generation to final disposal	1	2.9	2.9	44.1	
	From generation till treatment	1	2.9	2.9	47.1	
	Generator of hazardous waste is responsible to ensure the correct disposalaway from the community	1	2.9	2.9	50.0	
	Health care facility is responsible of waste from the point of generation to disposal	1	2.9	2.9	52.9	
	It's a process whereby waste is tracked from generation to disposa	1	2.9	2.9	55.9	
	It's a tracking system meaning that hazardous waste generators must track waste from the moment of inception as a hazardous material until the treatment and disposal of that hazardous waste.	1	2.9	2.9	58.8	
	management of waste from point of generation until disposal	1	2.9	2.9	61.8	
	management of waste from point of inception until destroyed	1	2.9	2.9	64.7	
	management of waste from the facility until it is treated	1	2.9	2.9	67.6	
	management of waste of point of generation until disposal	1	2.9	2.9	70.6	
	means all the seven steps followed to manage health care waste	1	2.9	2.9	73.5	
	point of generation to point of destruction	1	2.9	2.9	76.5	
	responsibility of managing waste from point of generation tofinal disposal	1	2.9	2.9	79.4	
	Taking care of the waste from point of generation until disposal	1	2.9	2.9	82.4	
	The generator of waste is responsible to ensure that waste is destroyed in accordance.	1	2.9	2.9	85.3	
	The generator of waste should take all necessary precautions to avoid soilages from souce until disposal	1	2.9	2.9	88.2	
	The gradle to grave principles requires waste to be segregated at point of generation, to be handled safe until point of incineration	1	2.9	2.9	91.2	
	The management of waste from the source to disposal in a manner that des not pose a threat or risk to human health and the environment	1	2.9	2.9	94.1	
	The steps/ pathway thatwaste follows from the point of generation up until its disposal	1	2.9	2.9	97.1	
	This ensuring that waste is handled safely or in a safe manner from point of generation to the ppint of disposal/ landfilling	1	2.9	2.9	100.0	
	Total	34	100.0	100.0		


4.2. Which of the following activities are seen as part of the “cradle to grave principle”?	
	Frequency	Percent	Valid Percent	Cumulative Percent	
Valid	1	1	2.9	2.9	2.9	
	1,2,3,4,5	25	73.5	73.5	76.5	
	1,2,3,5	1	2.9	2.9	79.4	
	1,2,4	1	2.9	2.9	82.4	
	1,2,4,5	2	5.9	5.9	88.2	
	1,4,5	1	2.9	2.9	91.2	
	1,5	1	2.9	2.9	94.1	
	2.5	1	2.9	2.9	97.1	
	3,4,5	1	2.9	2.9	100.0	
	Total	34	100.0	100.0		


4.2. Which of the following activities are seen as part of the “cradle to grave principle”? Generation at source level	
	Frequency	Percent	Valid Percent	Cumulative Percent	
Valid	Generation at source level	32	94.1	100.0	100.0	
Missing	System	2	5.9			
Total	34	100.0			


4.2. Which of the following activities are seen as part of the “cradle to grave principle”? Collection from source to storage area	
	Frequency	Percent	Valid Percent	Cumulative Percent	
Valid	Collection from source to storage area	30	88.2	100.0	100.0	
Missing	System	4	11.8			
Total	34	100.0			


4.2. Which of the following activities are seen as part of the “cradle to grave principle”? Storage-facility	
	Frequency	Percent	Valid Percent	Cumulative Percent	
Valid	Storage-facility	28	82.4	100.0	100.0	
Missing	System	6	17.6			
Total	34	100.0			


4.2. Which of the following activities are seen as part of the “cradle to grave principle”? Transportation of HCW	
	Frequency	Percent	Valid Percent	Cumulative Percent	
Valid	Transportation of HCW	30	88.2	100.0	100.0	
Missing	System	4	11.8			
Total	34	100.0			


4.2. Which of the following activities are seen as part of the “cradle to grave principle”? Treatment facility (e.g. incineration)	
	Frequency	Percent	Valid Percent	Cumulative Percent	
Valid	Treatment facility (e.g. incineration)	31	91.2	100.0	100.0	
Missing	System	3	8.8			
Total	34	100.0			


4.3. Indicate four environmental risks applicable to your HCF:	
	Frequency	Percent	Valid Percent	Cumulative Percent	
Valid	1,2,3,5,7	2	5.9	5.9	5.9	
	1,2,3,6,7	1	2.9	2.9	8.8	
	1,2,3,7	1	2.9	2.9	11.8	
	1,2,5,7	1	2.9	2.9	14.7	
	1,3,4,5,6,7	1	2.9	2.9	17.6	
	1,3,5,7	2	5.9	5.9	23.5	
	1,3,7	1	2.9	2.9	26.5	
	1,5,6,7	1	2.9	2.9	29.4	
	1.7	1	2.9	2.9	32.4	
	2,3,4	1	2.9	2.9	35.3	
	2,3,4,6	1	2.9	2.9	38.2	
	2,3,4,7	2	5.9	5.9	44.1	
	2,3,5	1	2.9	2.9	47.1	
	2,3,6,7	2	5.9	5.9	52.9	
	2,4,5,6,7	1	2.9	2.9	55.9	
	3,5	1	2.9	2.9	58.8	
	3,6,7	1	2.9	2.9	61.8	
	3.5	2	5.9	5.9	67.6	
	5	3	8.8	8.8	76.5	
	5,6,7	1	2.9	2.9	79.4	
	5.7	3	8.8	8.8	88.2	
	6	1	2.9	2.9	91.2	
	6.7	1	2.9	2.9	94.1	
	7	2	5.9	5.9	100.0	
	Total	34	100.0	100.0		


4.3. Indicate four environmental risks applicable to your HCF: Availability of electricity	
	Frequency	Percent	Valid Percent	Cumulative Percent	
Valid	Availability of electricity	11	32.4	100.0	100.0	
Missing	System	23	67.6			
Total	34	100.0			


4.3. Indicate four environmental risks applicable to your HCF: Global warming	
	Frequency	Percent	Valid Percent	Cumulative Percent	
Valid	Global warming	13	38.2	100.0	100.0	
Missing	System	21	61.8			
Total	34	100.0			


4.3. Indicate four environmental risks applicable to your HCF: Chemical pollution	
	Frequency	Percent	Valid Percent	Cumulative Percent	
Valid	Chemical pollution	19	55.9	100.0	100.0	
Missing	System	15	44.1			
Total	34	100.0			


4.3. Indicate four environmental risks applicable to your HCF: Radioactive pollution	
	Frequency	Percent	Valid Percent	Cumulative Percent	
Valid	Radioactive pollution	6	17.6	100.0	100.0	
Missing	System	28	82.4			
Total	34	100.0			


4.3. Indicate four environmental risks applicable to your HCF: Availability of clean water	
	Frequency	Percent	Valid Percent	Cumulative Percent	
Valid	Availability of clean water	18	52.9	100.0	100.0	
Missing	System	16	47.1			
Total	34	100.0			


4.3. Indicate four environmental risks applicable to your HCF: Toxic metals in the environment	
	Frequency	Percent	Valid Percent	Cumulative Percent	
Valid	Toxic metals in the environment	11	32.4	100.0	100.0	
Missing	System	23	67.6			
Total	34	100.0			


4.3. Indicate four environmental risks applicable to your HCF: Water pollution	
	Frequency	Percent	Valid Percent	Cumulative Percent	
Valid	Water pollution	23	67.6	100.0	100.0	
Missing	System	11	32.4			
Total	34	100.0			


4.4. How often does the HCF take samples of the effluent water?	
	Frequency	Percent	Valid Percent	Cumulative Percent	
Valid	Monthly	9	26.5	26.5	26.5	
	Quarterly (Every 3 months)	10	29.4	29.4	55.9	
	Bi-annually (Every 6 months)	2	5.9	5.9	61.8	
	Annually (Once a year)	1	2.9	2.9	64.7	
	Never	5	14.7	14.7	79.4	
	Unknown	7	20.6	20.6	100.0	
	Total	34	100.0	100.0		


4.5. Is the management of HCRW part of a formal management system? (E.g. International Organization for Standardization 14001or 9001, the Council for Health Service Accreditation for Southern Africa or the office of Health Standards Compliance etc.).	
	Frequency	Percent	Valid Percent	Cumulative Percent	
Valid	Yes	6	17.6	18.2	18.2	
	No	4	11.8	12.1	30.3	
	Uncertain	23	67.6	69.7	100.0	
	Total	33	97.1	100.0		
Missing	System	1	2.9			
Total	34	100.0			


4.6. If answered Yes to 4.5, what is the role of the management system within your HCF?	
	Frequency	Percent	Valid Percent	Cumulative Percent	
Valid		28	82.4	82.4	82.4	
	2	1	2.9	2.9	85.3	
	Identify and evaluate risks as a means to reduce injuries to patients, staff members and visitors within the facility. Also ensures the reduction of health care risk waste in the facility	1	2.9	2.9	88.2	
	The role of the management system within the facility is to ensure that correct waste management practices are followed and staff members receive adequate traning on waste management	1	2.9	2.9	91.2	
	To ensure segregation and comly with the Act	1	2.9	2.9	94.1	
	To ensure that health care waste is handled as according to the standards by providing correct waste consumables and personal pretective equipment that is of good quality and meets the standards	1	2.9	2.9	97.1	
	To ensure that use comply within the South African standard and international standards	1	2.9	2.9	100.0	
	Total	34	100.0	100.0		


4.7. Are there any formal conservation (saving) strategies for the following? Saving of electricity	
	Frequency	Percent	Valid Percent	Cumulative Percent	
Valid	Yes	6	17.6	17.6	17.6	
	No	23	67.6	67.6	85.3	
	Uncertain	5	14.7	14.7	100.0	
	Total	34	100.0	100.0		


4.7. Are there any formal conservation (saving) strategies for the following? Saving of water	
	Frequency	Percent	Valid Percent	Cumulative Percent	
Valid	Yes	5	14.7	14.7	14.7	
	No	26	76.5	76.5	91.2	
	Uncertain	3	8.8	8.8	100.0	
	Total	34	100.0	100.0		


4.7. Are there any formal conservation (saving) strategies for the following? Decrease of HCW	
	Frequency	Percent	Valid Percent	Cumulative Percent	
Valid	Yes	19	55.9	55.9	55.9	
	No	14	41.2	41.2	97.1	
	Uncertain	1	2.9	2.9	100.0	
	Total	34	100.0	100.0		


4.7. Are there any formal conservation (saving) strategies for the following? Recycling of paper/plastic/cardboard	
	Frequency	Percent	Valid Percent	Cumulative Percent	
Valid	Yes	24	70.6	70.6	70.6	
	No	10	29.4	29.4	100.0	
	Total	34	100.0	100.0		


4.8. If answered Yes to 4.7, please give detail of the strategies: Electricity	
	Frequency	Percent	Valid Percent	Cumulative Percent	
Valid		28	82.4	82.4	82.4	
	By switching off our offices appliances after work	1	2.9	2.9	85.3	
	don't leave lights on unncessary	1	2.9	2.9	88.2	
	usage of energy saving bulbs	1	2.9	2.9	91.2	
	using or installed long life energy saving light bulb	1	2.9	2.9	94.1	
	Using steam to heat water instead of a geyser	1	2.9	2.9	97.1	
	We ensure that all the computers and lights at the admissions and entire hospital are swithed off when knocking off	1	2.9	2.9	100.0	
	Total	34	100.0	100.0		


4.8. If answered Yes to 4.7, please give detail of the strategies: Water	
	Frequency	Percent	Valid Percent	Cumulative Percent	
Valid		29	85.3	85.3	85.3	
	closing the tap when not in use	1	2.9	2.9	88.2	
	Ensure that all the leakages are fixed to avoid water wastage	1	2.9	2.9	91.2	
	Ensuring that all leaking taps and pipes are fixed immediately	1	2.9	2.9	94.1	
	Ensuring that through out the facility there are no leaking taps, broken toilets and burst pipes that are left unattended	1	2.9	2.9	97.1	
	repair all leaking taps pipes and old plumbng system	1	2.9	2.9	100.0	
	Total	34	100.0	100.0		


4.8. If answered Yes to 4.7, please give detail of the strategies: HCW	
	Frequency	Percent	Valid Percent	Cumulative Percent	
Valid		15	44.1	44.1	44.1	
	better segregation	1	2.9	2.9	47.1	
	By doing training, eduvation by posters, walk abouts and stff training	1	2.9	2.9	50.0	
	By segregation of waste we decrease HCW	1	2.9	2.9	52.9	
	Employing EHPs permanately to monitor the generation of health care waste	1	2.9	2.9	55.9	
	formal and informal trainings. Road shows and awareness campaighns. Inspections and report discussions	1	2.9	2.9	58.8	
	monthly audits are done to ensure proper segregation	1	2.9	2.9	61.8	
	needs much more attention. On-going training and education	1	2.9	2.9	64.7	
	on-going training on a daily basis	1	2.9	2.9	67.6	
	Proper segregation and using only necessary materials during medical procedures.	1	2.9	2.9	70.6	
	Recyclig of vials and CSSD utensils	1	2.9	2.9	73.5	
	recycling	1	2.9	2.9	76.5	
	segregation of HCW is managed daily and traning	1	2.9	2.9	79.4	
	segregation of waste	1	2.9	2.9	82.4	
	Training are conducted at the facility	1	2.9	2.9	85.3	
	Training is provided tpo al staff on waste segregation, policies and regulations are used to enforce compliance	1	2.9	2.9	88.2	
	Training on proper waste segregation.	1	2.9	2.9	91.2	
	training to ensure proper segregation	1	2.9	2.9	94.1	
	Training, workshops and regular monitoring	1	2.9	2.9	97.1	
	waste managemnt is monitored to indicate if there is a decrease in health care waste.	1	2.9	2.9	100.0	
	Total	34	100.0	100.0		


4.8. If answered Yes to 4.7, please give detail of the strategies: Recycling	
	Frequency	Percent	Valid Percent	Cumulative Percent	
Valid		8	23.5	23.5	23.5	
	A service provider has been contracted for recycling and purchasing the goods	1	2.9	2.9	26.5	
	All cardboards are donated to Blind NGO at the community	1	2.9	2.9	29.4	
	By doing recycling of paper cardboard. We are reducing the waste in the facility	1	2.9	2.9	32.4	
	collected by a service provider	1	2.9	2.9	35.3	
	collected by recycling company. Paper shredded	1	2.9	2.9	38.2	
	Have community members recycle general waste	1	2.9	2.9	41.2	
	Impact recycling the papare and cardboard generated by the facility	1	2.9	2.9	44.1	
	In place but not functioning 100%-needs improvement and proper recycling containers	1	2.9	2.9	47.1	
	office paper and cardboxes are recycled	1	2.9	2.9	50.0	
	paper and cardbox generatedat the facility is recycled	1	2.9	2.9	52.9	
	Paper, cardboard boxes and plastics are being recycled	1	2.9	2.9	55.9	
	Recycle office paper	1	2.9	2.9	58.8	
	recycling at the storage area does take place	1	2.9	2.9	61.8	
	Recycling boxes for are placed in offices and points were office paper is used so as to ensure that staff members take part in the recycling programme	1	2.9	2.9	64.7	
	Recycling cardboard	1	2.9	2.9	67.6	
	Recycling company collects sorted waste for recycling	1	2.9	2.9	70.6	
	Recycling containers	1	2.9	2.9	73.5	
	Recycling initiative has been implemented to reduce wastegoing to the landfill	1	2.9	2.9	76.5	
	recycling is separated at the storage area	1	2.9	2.9	79.4	
	Recycling of boxes and paper	1	2.9	2.9	82.4	
	recycling paper	1	2.9	2.9	85.3	
	The facility keeps all the unwanted paper for recycling	1	2.9	2.9	88.2	
	The institution has contract with Nampac company wwhereby papares are collected	1	2.9	2.9	91.2	
	The office papers and plastic is collected by Impact company for recycling	1	2.9	2.9	94.1	
	We recycle paper in every office	1	2.9	2.9	97.1	
	we recycle paper, cardboard etc	1	2.9	2.9	100.0	
	Total	34	100.0	100.0		


4.9. Does your HCF have a HCRW service provider?	
	Frequency	Percent	Valid Percent	Cumulative Percent	
Valid	Yes	34	100.0	100.0	100.0	


4.10. If answered Yes to question 4.9, is the service provider in compliance with the following licences? Hazardous waste transporter licence from Gauteng Department of Agriculture and Rural Development	
	Frequency	Percent	Valid Percent	Cumulative Percent	
Valid	Yes	33	97.1	100.0	100.0	
Missing	System	1	2.9			
Total	34	100.0			


4.10. If answered Yes to question 4.9, is the service provider in compliance with the following licences? Transfer facility licence from  Gauteng Department of Agriculture and Rural Development	
	Frequency	Percent	Valid Percent	Cumulative Percent	
Valid	Yes	32	94.1	94.1	94.1	
	No	2	5.9	5.9	100.0	
	Total	34	100.0	100.0		


4.10. If answered Yes to question 4.9, is the service provider in compliance with the following licences? Waste treatment licence from Gauteng Department of Agriculture and Rural Development facility	
	Frequency	Percent	Valid Percent	Cumulative Percent	
Valid	Yes	33	97.1	97.1	97.1	
	5	1	2.9	2.9	100.0	
	Total	34	100.0	100.0		


4.10. If answered Yes to question 4.9, is the service provider in compliance with the following licences? Air emission licence from local municipality	
	Frequency	Percent	Valid Percent	Cumulative Percent	
Valid	Yes	34	100.0	100.0	100.0	


5.1. Is the HCF involved in community participation projects with regards to recycling any HCW?	
	Frequency	Percent	Valid Percent	Cumulative Percent	
Valid	Yes	14	41.2	41.2	41.2	
	No	15	44.1	44.1	85.3	
	Uncertain	5	14.7	14.7	100.0	
	Total	34	100.0	100.0		


5.2. If answered Yes to 5.1, which HCW is being recycled? Office Paper	
	Frequency	Percent	Valid Percent	Cumulative Percent	
Valid	Yes	15	44.1	93.8	93.8	
	No	1	2.9	6.3	100.0	
	Total	16	47.1	100.0		
Missing	System	18	52.9			
Total	34	100.0			


5.2. If answered Yes to 5.1, which HCW is being recycled? Glass	
	Frequency	Percent	Valid Percent	Cumulative Percent	
Valid	Yes	1	2.9	7.7	7.7	
	No	12	35.3	92.3	100.0	
	Total	13	38.2	100.0		
Missing	System	21	61.8			
Total	34	100.0			


5.2. If answered Yes to 5.1, which HCW is being recycled? Plastic	
	Frequency	Percent	Valid Percent	Cumulative Percent	
Valid	Yes	3	8.8	23.1	23.1	
	No	10	29.4	76.9	100.0	
	Total	13	38.2	100.0		
Missing	System	21	61.8			
Total	34	100.0			


5.2. If answered Yes to 5.1, which HCW is being recycled? Aluminium Cans	
	Frequency	Percent	Valid Percent	Cumulative Percent	
Valid	Yes	3	8.8	21.4	21.4	
	No	11	32.4	78.6	100.0	
	Total	14	41.2	100.0		
Missing	System	20	58.8			
Total	34	100.0			


5.2. If answered Yes to 5.1, which HCW is being recycled? Newspaper/ Magazines	
	Frequency	Percent	Valid Percent	Cumulative Percent	
Valid	Yes	4	11.8	28.6	28.6	
	No	10	29.4	71.4	100.0	
	Total	14	41.2	100.0		
Missing	System	20	58.8			
Total	34	100.0			


5.3. Does the HCF recognise the waste reclaimers?	
	Frequency	Percent	Valid Percent	Cumulative Percent	
Valid	Yes	6	17.6	20.0	20.0	
	No	16	47.1	53.3	73.3	
	Uncertain	8	23.5	26.7	100.0	
	Total	30	88.2	100.0		
Missing	System	4	11.8			
Total	34	100.0			


5.4. If answered Yes to 5.3, has the waste reclaimers received any training?	
	Frequency	Percent	Valid Percent	Cumulative Percent	
Valid	Yes	2	5.9	22.2	22.2	
	No	2	5.9	22.2	44.4	
	Uncertain	5	14.7	55.6	100.0	
	Total	9	26.5	100.0		
Missing	System	25	73.5			
Total	34	100.0			


5.5. How does your HCF manage HCRW issued by your HCF that is generated from home? (E.g. needles, syringes and test strips from a diabetes patient, unused or expired medication and dialysis bag from a peritoneal dialysis patient etc.).	
	Frequency	Percent	Valid Percent	Cumulative Percent	
Valid		5	14.7	14.7	14.7	
	Advice patients to bring back waste to pharmacy or deliver it to their nearest clinic	1	2.9	2.9	17.6	
	All diabetic containers are returned to the health care facility everytime the patients returns for medication or check-up. Patients are also adviced to return expired medication to the facility	1	2.9	2.9	20.6	
	All patients who are on insulin are adviced to bring back their health care risk watse to the facility for proper disposal. There is a register which the facility use to ensure the system being followed	1	2.9	2.9	23.5	
	Diabetic patients are encouraged to return their 1.4 L sharps containers to the facility	1	2.9	2.9	26.5	
	Diabetic patients are give 1.4 L sharps containers whenthey collect medication from pharmacy. The person issuing medication the gives eduv=cation on how the patient should disposed needle and when should they return the container to the health establishment.	1	2.9	2.9	29.4	
	Diabetic patients receive small containers for sharps which is used in a register with name, cell no, adress when seaked and returned date are written on container and it is placed in the storage area for collection	1	2.9	2.9	32.4	
	diabetic patients return their used needles to the facility	1	2.9	2.9	35.3	
	During pharmacy week, the patients are adviced to bring their expired medications to the facility	1	2.9	2.9	38.2	
	Education on the return pf the used needles is given to the patients	1	2.9	2.9	41.2	
	In accordance to the SOP that states that sharp containers should be provided to the diabetic patient and returned when the next appointment is due	1	2.9	2.9	44.1	
	Insulin syringes taken back to pharmacy and dispose in sharp container and used medication, expired medication put in green containers or death of patient	1	2.9	2.9	47.1	
	needles are brought back by the diabetes patinets	1	2.9	2.9	50.0	
	Needles are taken back to pharmacy by means of the provided 1.4 L shraps container	1	2.9	2.9	52.9	
	Patients are requested to bring back used medication, syringes and needls when collecting new medication. Record is kept for all medication and equipment taken by patient	1	2.9	2.9	55.9	
	patients are required to return their used needls/expired medication or unused medication in order to receivenew medication.	1	2.9	2.9	58.8	
	patients are told to return their sharps container when they collect their monthly medication. This is only for diabetic patients.	1	2.9	2.9	61.8	
	Patients are trained and are issued with diabetic sharps containers which they return when either full or have reached 90 days. Expired medication is brough back to the pharmacy when expired for proper disposal	1	2.9	2.9	64.7	
	patients bring back their used needles	1	2.9	2.9	67.6	
	Patients bring the container back to the facility for disposal	1	2.9	2.9	70.6	
	Patients brings back expired medication back in the hospital and diabetic patients bring back the needles in the hospital, Dialysis patients takes the waste to their nearest clinics	1	2.9	2.9	73.5	
	patients return the used needles	1	2.9	2.9	76.5	
	Patients with expired medication are encouraged to return the medication to the facility and it is didposed in the pharmaceutical contianer. Diabetic patients are provided with a sharp container which they return when full	1	2.9	2.9	79.4	
	sharps containers filled with needles are returned by patients	1	2.9	2.9	82.4	
	The patients are educated to return the used needles in the provided container	1	2.9	2.9	85.3	
	The patients bring in the waste and it is put in the relevant containers/plastic and handled according to our procedure e.g. sharps will be put in a yellow container as there asre and put in the sluice room to be collected by health care rislk collectors	1	2.9	2.9	88.2	
	There is a recording book to monitor such waste	1	2.9	2.9	91.2	
	They are disposed in different coloir coded containers and patients are trained on how to use and dispose after use as well as informed on the importance of bringing them back to the facility	1	2.9	2.9	94.1	
	used needles are returned to the facility after use	1	2.9	2.9	97.1	
	Yes, diabetic patients are being provided with 1.4 L sharps containers	1	2.9	2.9	100.0	
	Total	34	100.0	100.0		


5.6. In your opinion, which governmental organisation should ensure HCW is managed within the HCF?	
	Frequency	Percent	Valid Percent	Cumulative Percent	
Valid	1	15	44.1	44.1	44.1	
	1,2,3,4,5	1	2.9	2.9	47.1	
	1,3	1	2.9	2.9	50.0	
	1,3,4,5	1	2.9	2.9	52.9	
	1.3	6	17.6	17.6	70.6	
	2	2	5.9	5.9	76.5	
	2.4	1	2.9	2.9	79.4	
	3	5	14.7	14.7	94.1	
	4	1	2.9	2.9	97.1	
	5	1	2.9	2.9	100.0	
	Total	34	100.0	100.0		


5.6. In your opinion, which governmental organisation should ensure HCW is managed within the HCF? National Department of Health	
	Frequency	Percent	Valid Percent	Cumulative Percent	
Valid	National Department of Health	24	70.6	100.0	100.0	
Missing	System	10	29.4			
Total	34	100.0			


5.6. In your opinion, which governmental organisation should ensure HCW is managed within the HCF? Gauteng Department of Agriculture and Rural Development facility	
	Frequency	Percent	Valid Percent	Cumulative Percent	
Valid	Gauteng Department of Agriculture and Rural Development facility	4	11.8	100.0	100.0	
Missing	System	30	88.2			
Total	34	100.0			


5.6. In your opinion, which governmental organisation should ensure HCW is managed within the HCF? Department of Environmental Affairs	
	Frequency	Percent	Valid Percent	Cumulative Percent	
Valid	Department of Environmental Affairs	14	41.2	100.0	100.0	
Missing	System	20	58.8			
Total	34	100.0			


5.6. In your opinion, which governmental organisation should ensure HCW is managed within the HCF? Metros/ Municipality	
	Frequency	Percent	Valid Percent	Cumulative Percent	
Valid	Metros/ Municipality	4	11.8	100.0	100.0	
Missing	System	30	88.2			
Total	34	100.0			


5.6. In your opinion, which governmental organisation should ensure HCW is managed within the HCF? Gauteng Department of Health	
	Frequency	Percent	Valid Percent	Cumulative Percent	
Valid	Gauteng Department of Health	3	8.8	100.0	100.0	
Missing	System	31	91.2			
Total	34	100.0			
